# Author Correction: Long non-coding RNA *NR2F1-AS1* induces breast cancer lung metastatic dormancy by regulating NR2F1 and ΔNp63

**DOI:** 10.1038/s41467-021-26292-x

**Published:** 2021-10-07

**Authors:** Yingjie Liu, Peiyuan Zhang, Qiuyao Wu, Houqin Fang, Yuan Wang, Yansen Xiao, Min Cong, Tingting Wang, Yunfei He, Chengxin Ma, Pu Tian, Yajun Liang, Lun-Xiu Qin, Qingcheng Yang, Qifeng Yang, Lujian Liao, Guohong Hu

**Affiliations:** 1 CAS Key Laboratory of Tissue Microenvironment and Tumor, Shanghai Institute of Nutrition and Health, University of Chinese Academy of Sciences, Chinese Academy of Sciences, Shanghai, China; 2 Shanghai Institute of Nutrition and Health, Shanghai Jiao Tong University School of Medicine (SJTUSM) & Chinese Academy of Sciences, Shanghai, China; 3 Shanghai Key Laboratory of Regulatory Biology, School of Life Sciences, East China Normal University, Shanghai, China; 4 Department of General Surgery, Huashan Hospital, Fudan University, Shanghai, China; 5 Department of Orthopedics, Shanghai Jiao Tong University Affiliated Sixth People’s Hospital, Shanghai, China; 6 Department of Breast Surgery, Qilu Hospital of Shandong University, Ji’nan, China

**Keywords:** Breast cancer, Cancer stem cells, Metastasis, Tumour heterogeneity

Correction to: *Nature Communications* 10.1038/s41467-021-25552-0, published online 02 September 2021.

This Article contained an error in the Acknowledgments section. In the Acknowledgements section the author omitted to include a grant from the Ministry of Science and Technology of China, 2020YFA0112302, for which the work received financial support.

This error has been corrected in the HTML and PDF versions of the Article.

