# Supplementary material for: Long non-coding RNA NR2F1-AS1 induces breast cancer lung metastatic dormancy by regulating NR2F1 and ΔNp63
Source: Nat Commun. 2021 Sep 2;12:5232. doi: 10.1038/s41467-021-25552-0 (PMC8413371; doi:10.1038/s41467-021-25552-0)
Supplement: Supplementary file 1 — Supplementary_information [file 41467_2021_25552_MOESM1_ESM.pdf]

Supplementary Information

Supplementary Information contains 12 Supplementary Figures and 6 Supplementary Tables.

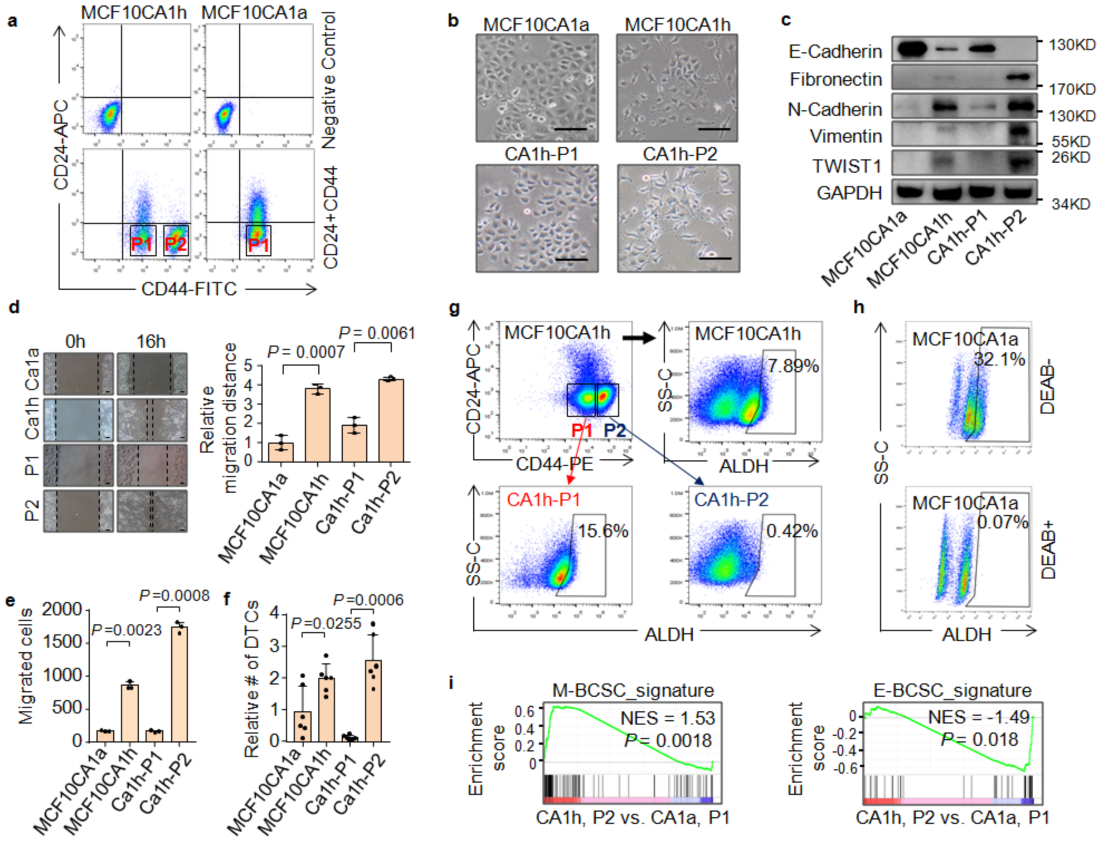

Supplementary Figure 1. P1 and P2 of MCF10CA1h are epithelial and mesenchymal-like subpopulations. (**a**) MCF10CA1h and MCF10CA1a cell subpopulations defined by CD24 and CD44 expression. (**b**) Representative images of cell morphology of MCF10 cell lines. Scale bar, 100 μm. Random microscopic field (*n* > 3) showed similar results. (**c**) Protein levels of EMT markers in MCF10 cell lines. (**d**) Wound healing of MCF10CA1a, MCF10CA1h, CA1h-P1 and CA1h-P2 (*n* = 3 independent experiments). Scale bar, 100 μm. (e) Number of transwell migration cells (*n* = 3 independent experiments). Scale bar, 100 μm. (**f**) Relative quantitation of GFP^+ DTCs in the blood of mice with orthotopic tumors of indicated breast cancer cells (n = 6 mice per group). (**g**) Flow cytometric analyses CD24/CD44 and ALDH expression of MCF10CA1h and its subpopulations. (**h**) The percentage of $ALDH^+$ cells in MCF10CA1a. (**i**)

GSEA of the M-BCSC and E-BCSC signatures with transcriptomic profiles of MCF10CA1h, CA1h-P2 versus MCF10CA1a, CA1h-P1. Data represent mean ± SEM (**d, e, f**). Statistical significance was determined by two-tailed unpaired *t*-test. Experiments in **c**, **g**, **h** were repeated at least three times independently with similar results; data from one representative experiment are shown.

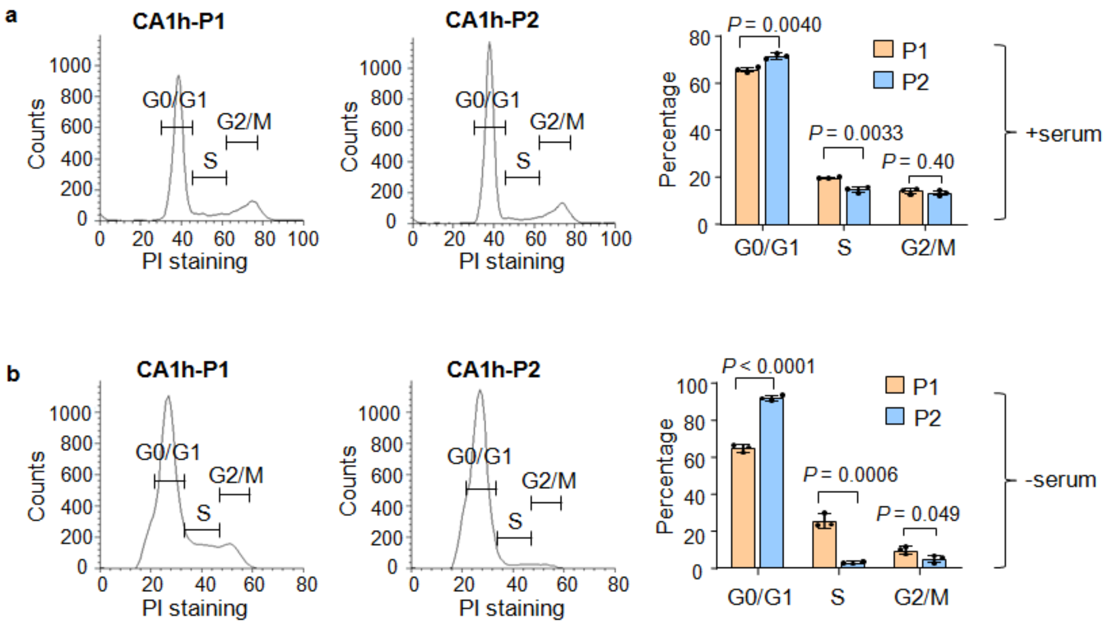

Supplementary Figure 2. *In vitro* proliferation analyses of P1 and P2 cells. Shown are cell cycle flow cytometry analyses of CA1h-P1 and CA1h-P2 in serum-supplemented (**a**) or serum-free (**b**) culture conditions (*n* = 3 independent experiments). Growth factors were supplemented in serum-free medium. Data represent mean ± SD. Statistical significance was determined by two-tailed unpaired *t*-test.

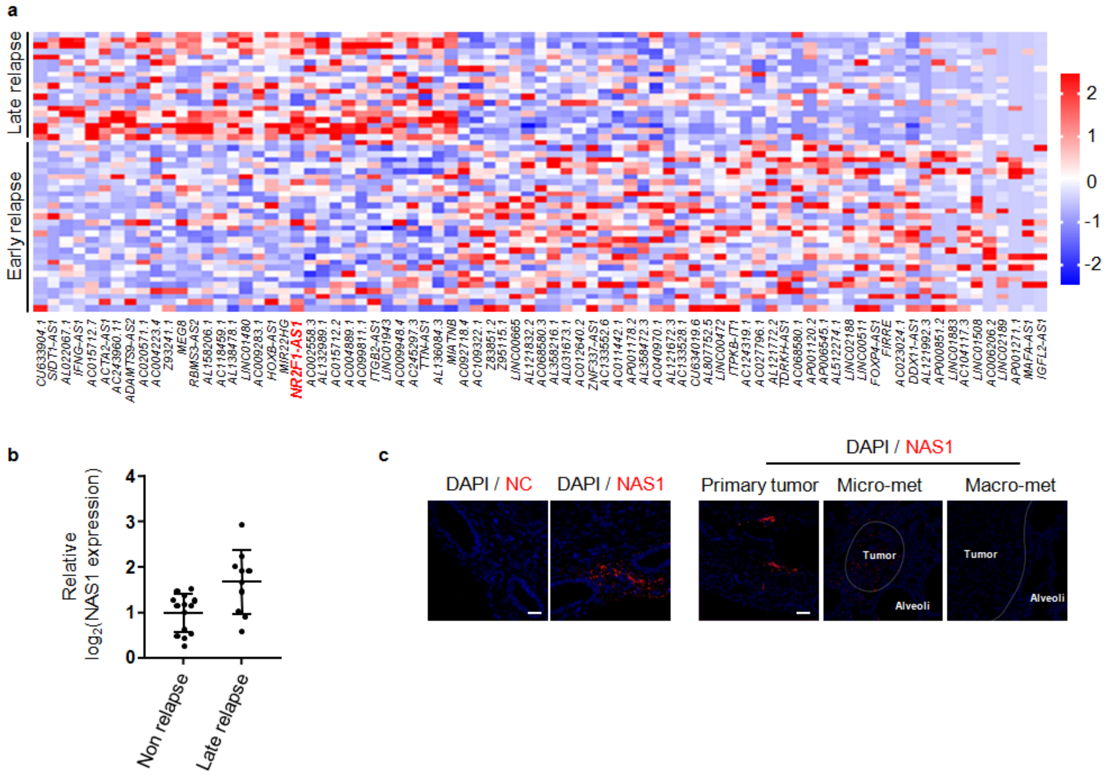

Supplementary Figure 3. *NAS1* expression in different stages of metastasis. (**a**) *NAS1* was upregulated in late-recurring versus early-recurring breast cancer patients. Shown are heatmap of differentially expressed lncRNAs in triple-negative breast cancer patients that recurred after >2 years following diagnosis (late relapse) versus patients that recurred within 2 years following diagnosis (early relapse) of the Fudan cohort[1] (*n* = 49 patients). (**b**) *NAS1* expression in ER^+ breast tumors[41] that recurred in 10 years after diagnosis (late relapse, *n* = 10 patients) versus those did not recur (no relapse, *n* = 14 patients). (**c**) FISH analyses of *NAS1* expression in primary tumors and different stages of metastasis of MCF10CA1h cells. Shown are the staining with *NAS1* probe and negative control (NC) probe (left), and *NAS1* expression primary tumors, micro-metastases (micro-met) and macro-metastases (macro-met) in lungs by MCF10CA1h cells (right). Scale bar, 100 μm. Data represent mean ± SD (**b**).

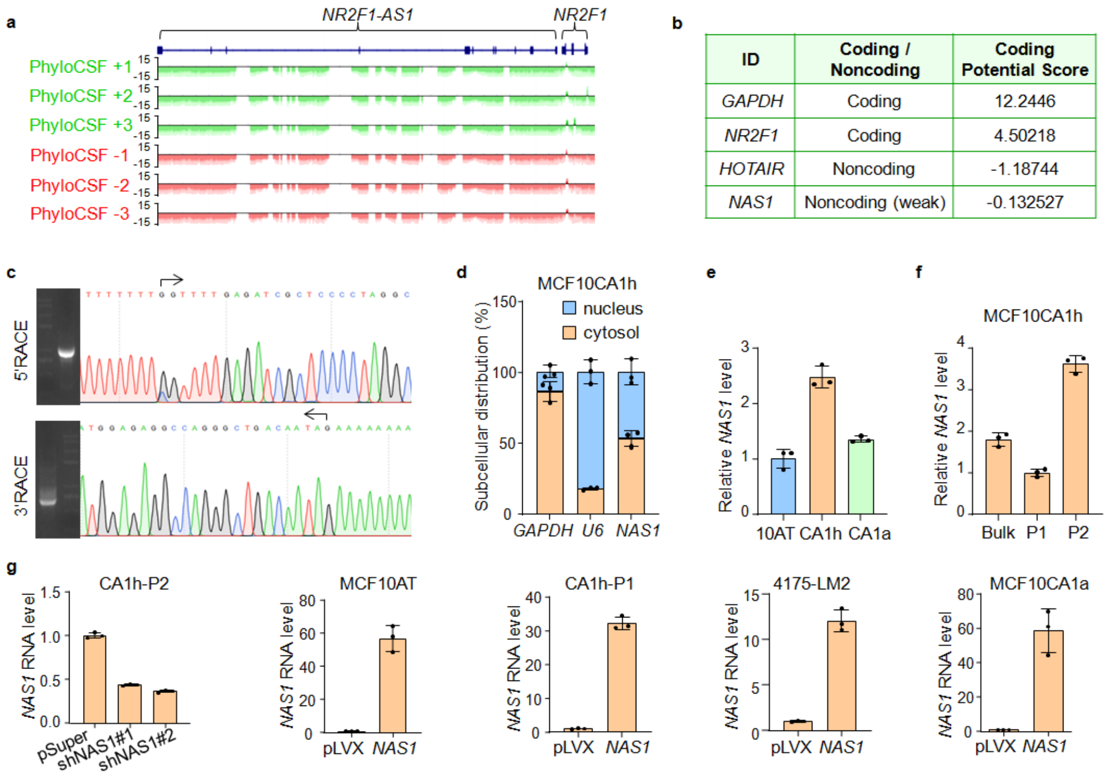

Supplementary Figure 4. Sequence and expression characterization of *NAS1*. (**a**) The coding abilities of *NAS1* and *NR2F1* analyzed with PhyloCSF of UCSC database. Green and red rows represent the coding ability of three different reading frames within the sense and antisense strands, respectively. Negative values indicate no coding potential. (**b**) The coding potential of *NAS1* scored by Coding Potential Calculator. Coding genes *GAPDH*, *NR2F1* and the lncRNA HOTAIR were used as control. (**c**) 5' and 3' RACE assays of *NAS1*. Arrows indicate the ends of RNA molecules. (**d**) Nucleus-cytosol distribution of *NAS1* in MCF10CA1h (n = 3 independent experiment). (**e, f**) qPCR verification of *NAS1* expression in different cell lines (n = 3 independent experiments). (**g**) *NAS1* expression after overexpression or knockdown in indicated cell lines (n = 3 replicates from one experiment). Data represent mean ± SD.

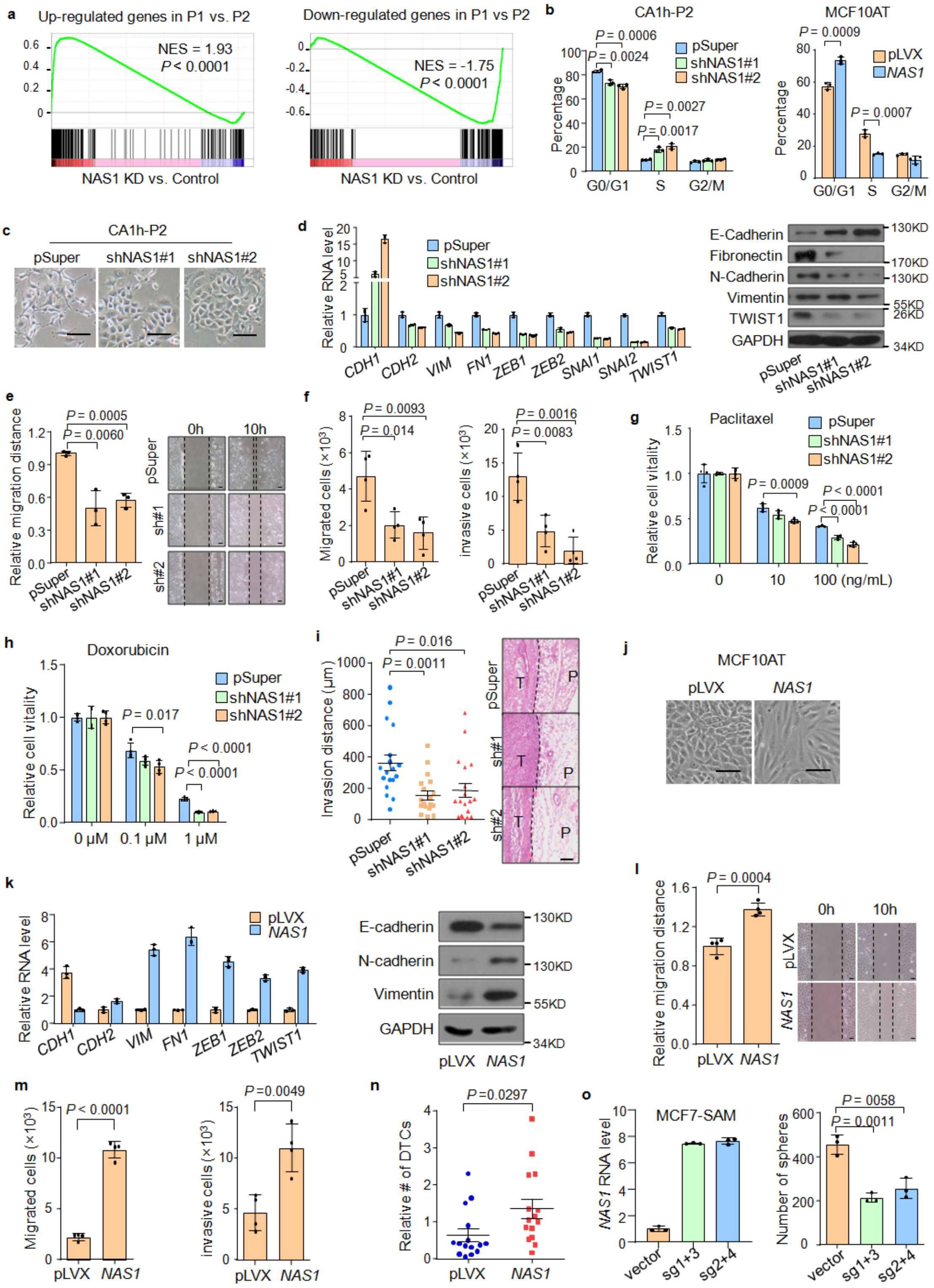

Supplementary Figure 5. *NAS1* promotes EMT, migration and invasion but inhibits tumor-initiating capability of cancer cells. (**a**) GSEA analyses of control vs. *NAS1* knockdown of CA1h-P2 cells with the up- (left) or down-regulated (right) gene sets in CA1h-P1 vs. CA1h-P2 cells. (**b**) Cell cycle analyses of CA1h-P2 with *NAS1* knockdown (left) and MCF10AT with *NAS1* overexpression (right) in serum-free cultures (n = 3 independent experiments).

(**c-f**) Representative images of cellular morphology (**c**), RNA and protein levels of EMT markers (n = 3 independent experiments) (**d**), wound healing (**e,** *n* = 3 independent experiments), transwell migration and invasion (**f**, *n* = 4 culturing experiments) of CA1h-P2 cells after *NAS1* knockdown. (**g, h**) MTT assays of CA1h-P2 with *NAS1* knockdown after paclitaxel (*n* = 4 wells from one experiment) (**g**) and doxorubicin (*n* = 4 wells from one experiment) (**h**) treatment in various concentrations. (**i**) H&E staining of tumor edges of orthotopic xenografts of CA1h-P2 with *NAS1* knockdown in mice (*n* = 18 RMFs from 6 tumors). T, tumor; P, para-carcinoma; dashed lines indicate tumor edges. (**j-m**) Representative images of cellular morphology (**j**), RNA and protein levels of EMT markers (*n* = 3 independent experiments) (**k**), wound healing (**l**, *n* = 4 independent experiments), transwell migration and invasion (**m**, *n* = 4 culturing experiments) of MCF10AT cells after *NAS1* overexpression. (**n**) Relative quantitation of GFP^+ DTCs in the blood of mice after orthotopic injection of MCF10CA1a cells with *NAS1* overexpression (*n* = 15 blood samples from 5 mice). (**o**) The *NAS1* RNA level (left) and the number of tumorspheres (right, *n* = 3 culturing experiments) after CRISPR/Cas9 synergistic activation mediator (SAM) activation of *NAS1* expression. Random microscopic field (*n* > 3) showed similar results in (**c**, **j**), respectively. The sequences of single guide RNAs (sgRNAs) used in this assay are provided in Supplementary Tables 5. Data represent mean ± SEM (**i**, **n**) or mean ± SD (others). Statistical significance was determined by two-tailed unpaired *t*-test. Scale bar, 100 μm.

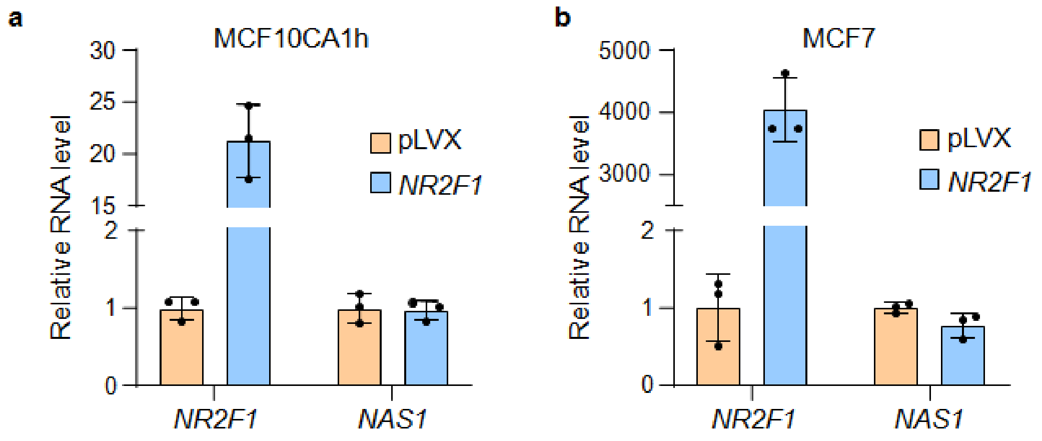

Supplementary Figure 6. *NR2F1* overexpression has no effect on *NAS1* expression. (**a**) *NAS1* expression in MCF10CA1h with *NR2F1* overexpression (n = 3 independent experiments). (**b**) *NAS1* expression in MCF7 with *NR2F1* overexpression (n = 3 independent experiments). Data represent mean ± SD.

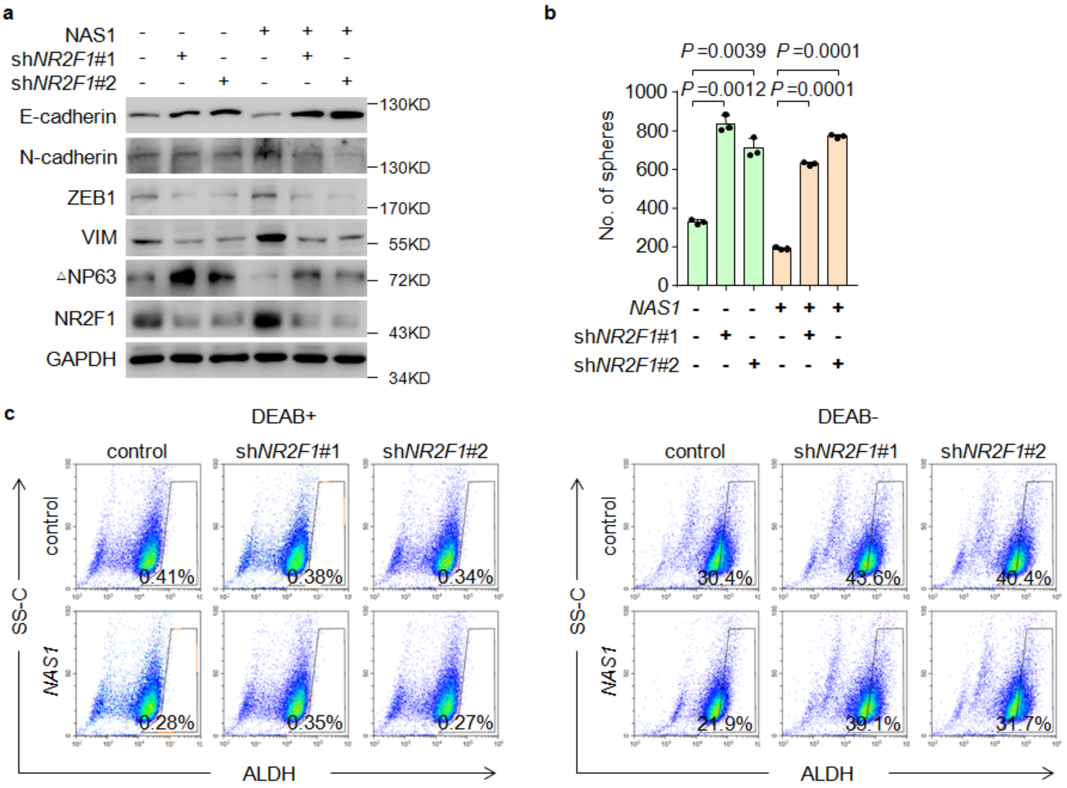

Supplementary Figure 7. *NR2F1* knockdown impairs the effects of *NAS1* on EMT and CSC properties. Shown are protein levels of EMT markers (**a**), tumorsphere formation (**b**, n = 3 culturing experiments), and flow cytometric analyses of the $ALDH^{+}$ BCSCs (**c**) in MCF10CA1a cells with *NAS1* overexpression and/or *NR2F1* knockdown. Data represent mean ± SEM (**b**). Statistical significance was determined by two-tailed unpaired *t*-test (**b**). Experiments in **a**, **c** were repeated at least three times independently with similar results; data from one representative experiment are shown.

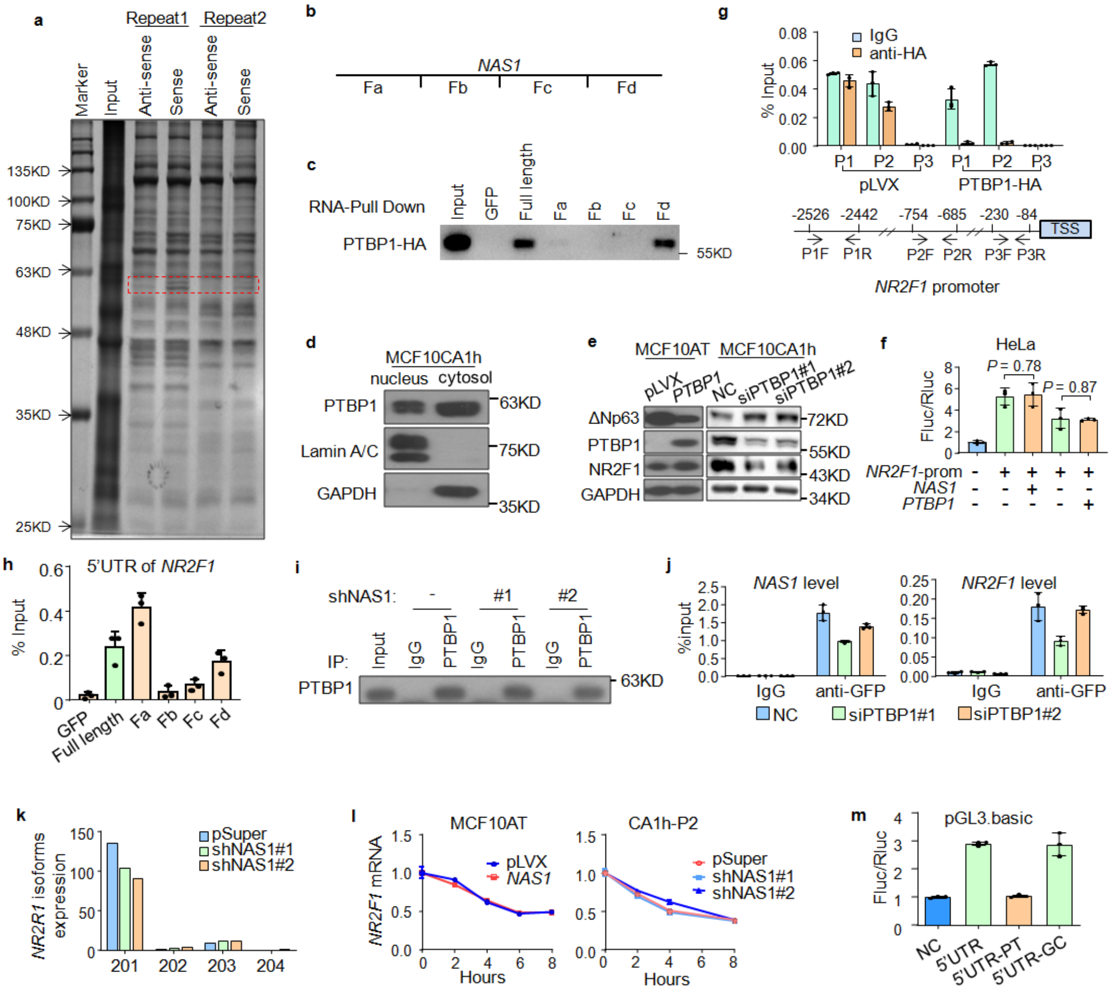

Supplementary Figure 8. *NAS1* and PTBP1 do not affect transcription, alternative splicing and RNA stability of *NR2F1*. (**a**) Representative image of silver staining of proteins interacting with *NAS1* in the RNA pull down assay using MCF10CA1h lysate. Red box circled the differential protein band analyzed by MS. RNA-pull down and silver staining were performed three times independently with similar results. (**b**) Schematic of the *NAS1* truncations. (**c**) Representative result of PTBP1 pulled down by *NAS1* truncations in HEK293T lysates. Pull-down were performed three times with similar results. (**d**) Representative result of nucleus-cytosol distribution of PTBP1 in MCF10CA1h. Nucleus-cytosol separations were performed three times with similar results. Lamin A/C and GAPDH were used as nuclear and cytosol markers, respectively. (**e**) NR2F1 and ΔNp63 expression after *PTBP1* overexpression or knockdown. NC, Negative Control. (**f**) *NR2F1* promoter activity after *NAS1* or *PTBP1* overexpression (n = 3 wells for each experiment).

(**g**) ChIP-qPCR analyses of the binding of PTBP1 to *NR2F1* promoter in HeLa (*n* = 3 replicates for each experiment). P1-3, primer pairs #1-3. (**h**) RIP assays of the binding of *NR2F1* 5'UTR mRNA to *NAS1* full length or various truncations in HEK293T lysates (*n* = 3 independent assays). (**i**) PTBP1 precipitated in the RIP assay of Fig. 5F. (**j**) The precipitated *NAS1* (left) and *NR2F1* mRNA (right) levels in the RIP assays of Fig. 5G (*n* = 3 replicates from one experiment). (**k**) Expression levels of *NR2F1* splicing variants analyzed in transcriptomic sequencing. Variant 201 is the coding variant and others are non-coding variants. (**l**) The stability of *NR2F1* mRNA in the indicated cells with *NAS1* overexpression or knockdown, treated with 5 μg/mL actinomycin D (*n* = 2 replicates from one experiment). (**m**) Promoter activity of *NR2F1*-5'UTR (*n* = 3 wells for each experiment). Data represent mean ± SD. Statistical significance was determined by two-tailed unpaired *t*-test.

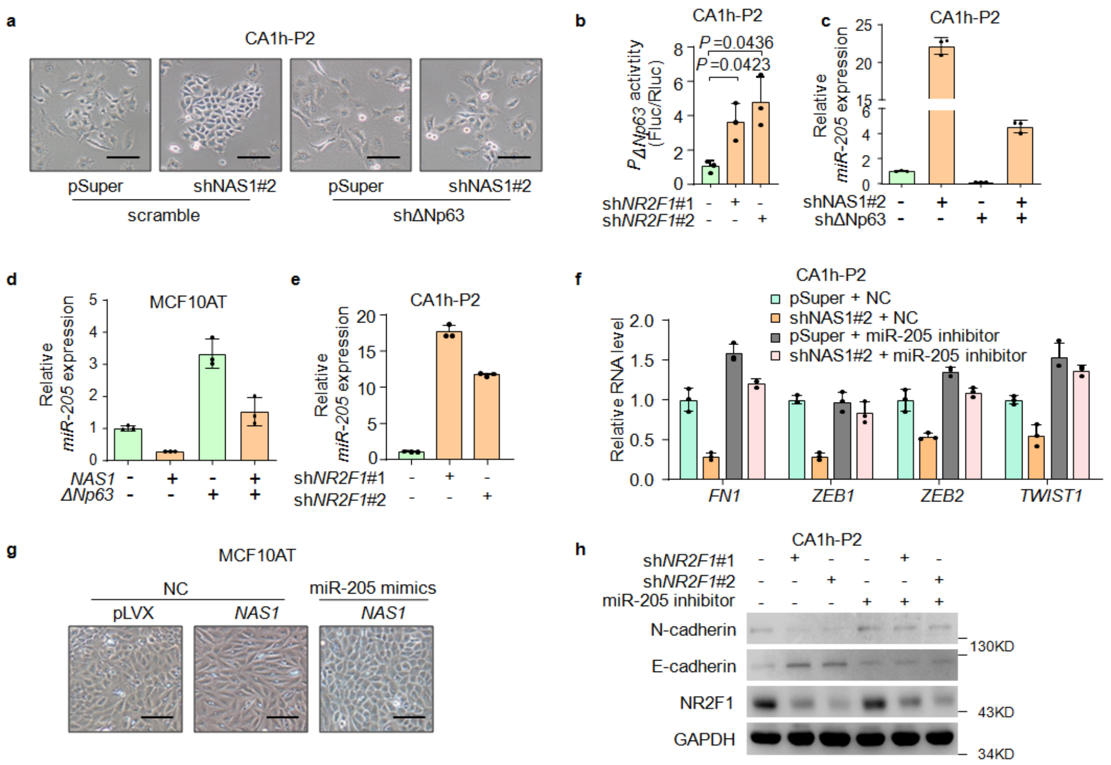

Supplementary Figure 9. Suppression of ΔNp63-miR-205 axis mediates the function of *NAS1* in EMT. (**a**) Morphology of CA1h-P2 cells with *NAS1* and/or *ΔNp63* knockdown. (**b**) Dual-luciferase reporter analysis of *ΔNp63* promoter activity in CA1h-P2 cells after *NR2F1* knockdown (n = 3 independent assays). (**c**) Expression of miR-205 in CA1h-P2 with *NAS1* and/or *ΔNp63* knockdown (n = 3 replicates from one experiment). (**d**) Expression of miR-205 in MCF10AT with *NAS1* and/or *ΔNp63* overexpression (n = 3 replicates from one experiment). (**e**) miR-205 expression of in CA1h-P2 after *NR2F1* knockdown (n = 3 replicates from one experiment). (**f**) EMT marker expression in CA1h-P2 with *NAS1* knockdown and/or miR-205 inhibitor (n = 3 replicates from one experiment). (**g**) Morphology of *NAS1*-overexpressing MCF10AT transfected with miR-205 mimics. NC, Negative Control. (**h**) EMT marker expression in CA1h-P2 with *NR2F1* knockdown and/or miR-205 inhibitor. Data represent mean ± SD. Scale bar, 100 μm. Statistical significance was determined by two-tailed unpaired *t*-test (**b**). Experiments in **a**, **g**, **h** were repeated at least three times independently with similar results; data from one representative experiment are shown.

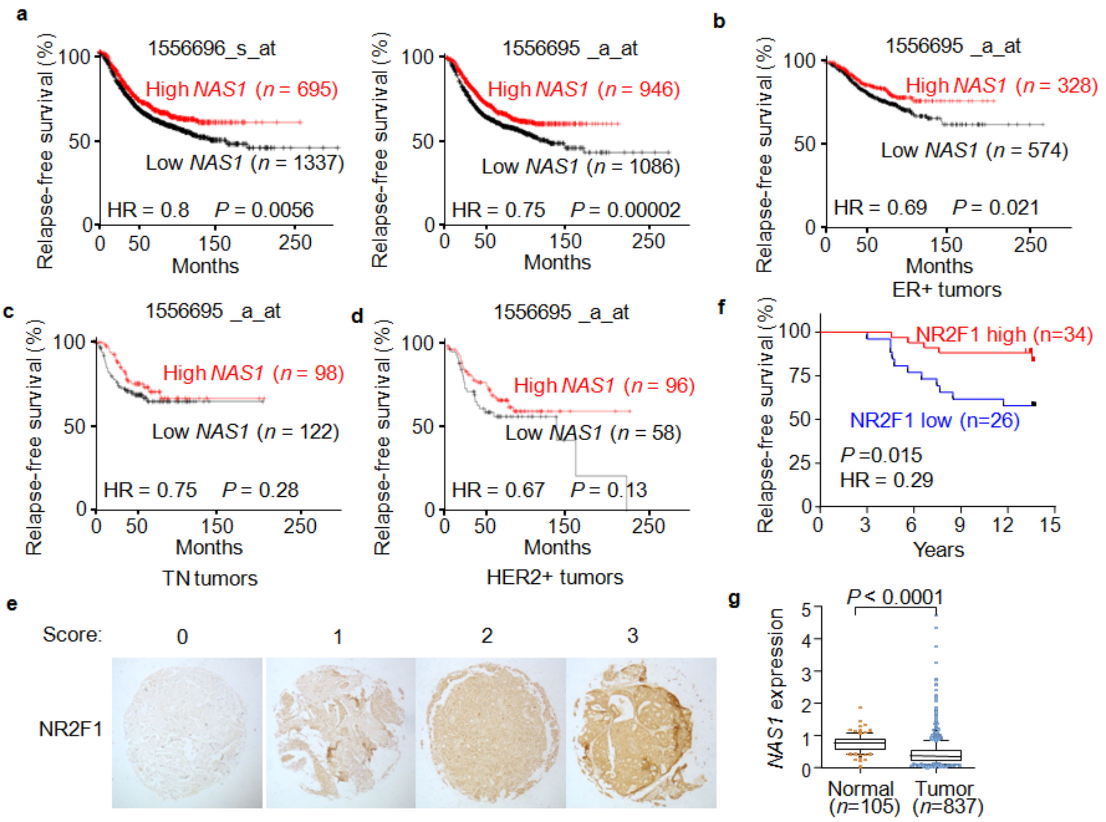

Supplementary Figure 10. The clinical relevance of *NAS1* and NR2F1 expression in human breast tumors. (**a-d**) Relapse-free survival analyses of the Kaplan-Meier Plotter[4] breast cancer cohort by *NAS1* expression. Shown are the analyses with both *NAS1* probes, 1556696_s_at and 1556695_a_at, of the overall cohort (**a**) and in different breast cancer subtypes (**b-d**). HR, hazard ratio. TN, Triple Negative. (**e-f**) Relapse-free survival analyses of NR2F1 expression by immunostaining of a breast cancer tissue microarray. The samples were scored to 0 (negative), 1 (weak), 2 (medium), 3 (strong) according to NR2F1 staining intensity (**e**), followed by survival analysis of the samples with low (scores 0 or 1) or high (scores 2 or 3) NR2F1 expression (**f**). (**g**) *NAS1* expression in normal breast tissues and tumors from the TCGA database. The center line indicates the median, the lower bound of the box indicates the 25th percentile, the upper bound of the box represent the 75th percentile, the lower whisker extends from the 25th percentile to 10th percentile, and the upper whisker extends from the 75th percentile to the 90th percentile. Statistical significance was determined by two-sided log-rank test (**a-d, f**) or two-tailed unpaired *t*-test (**g**).

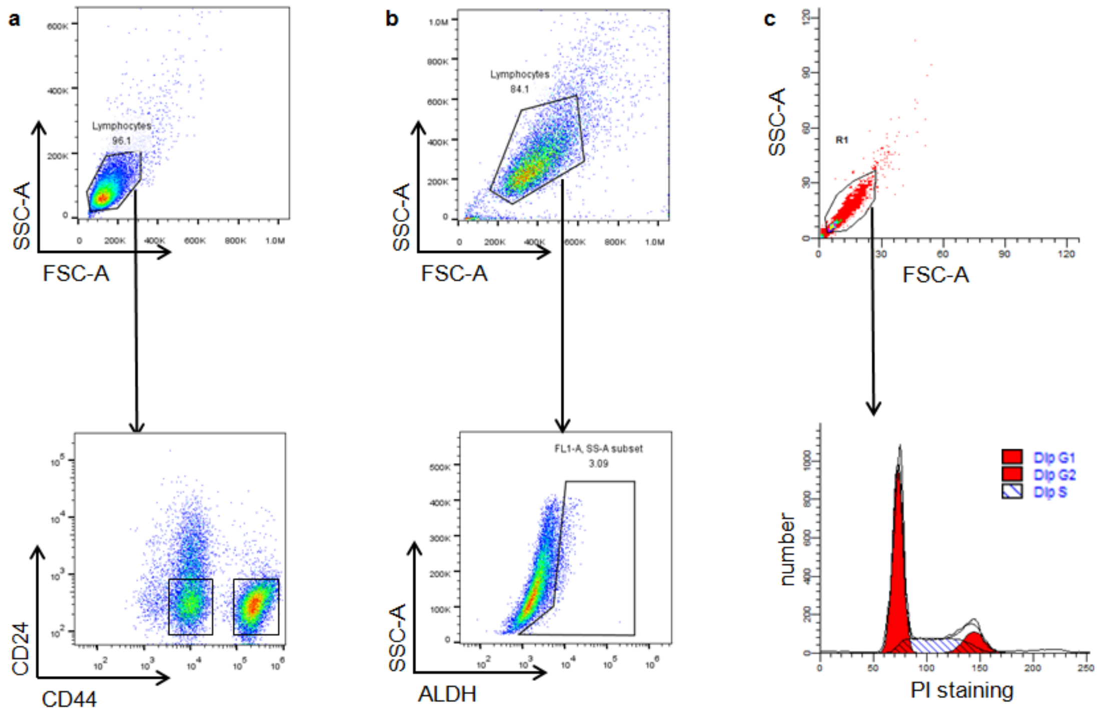

Supplementary Figure 11. Gating strategy of flow cytometric analyses.

Gate strategy of CD44^{+}CD24^{-} (**a**) was used in fig. 3b-c and supplementary fig. 1g. Gate strategy of ALDH^{+} (**b**) was used in fig. 3d-e, 4e, and supplementary fig. 1g-h, 7c. Cell cycle analysis (**c**) was used in supplementary fig. 2a-b.

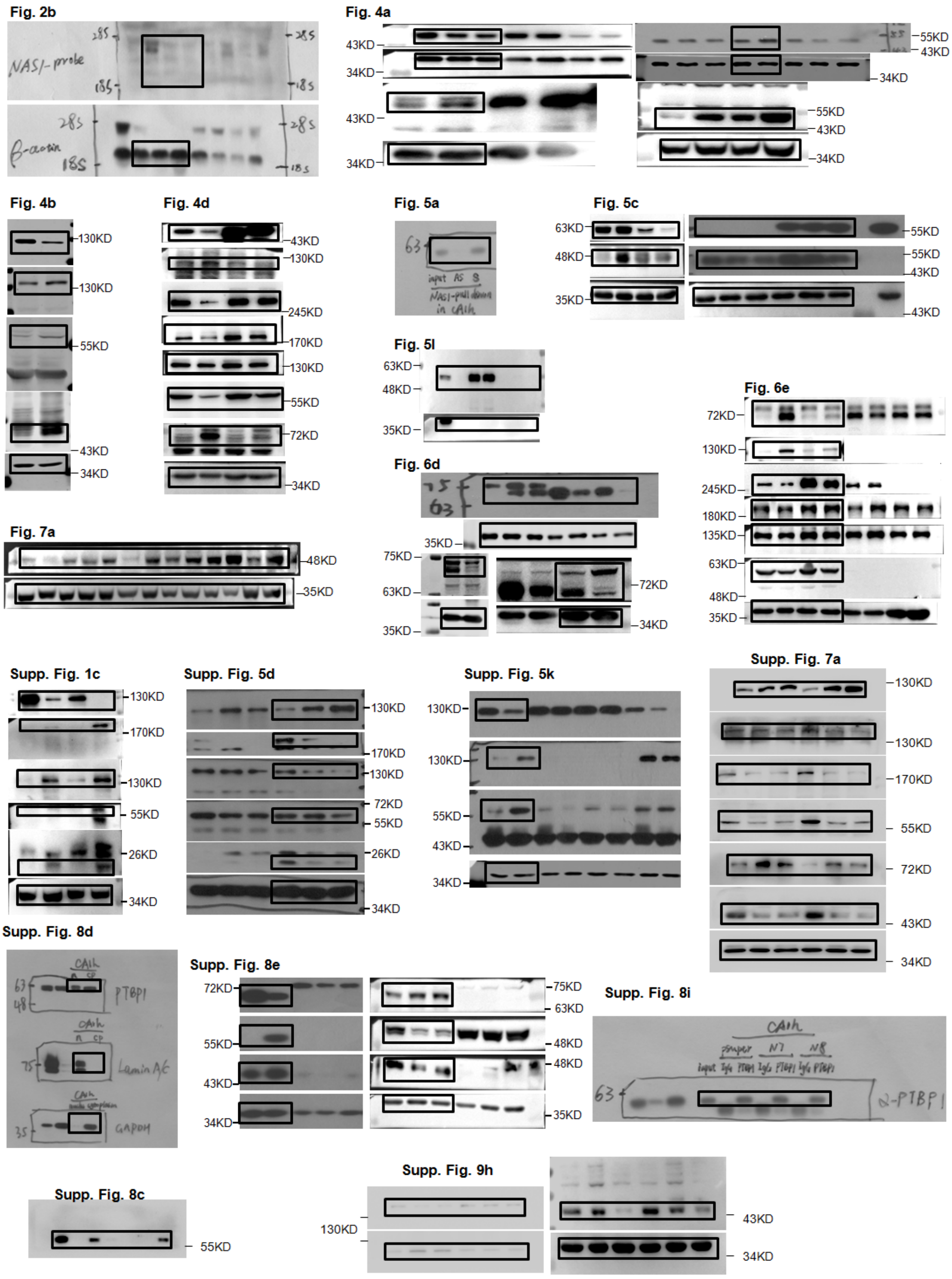

Supplementary Figure 12. Original uncropped Western blots in main and supplementary figures.

Supplementary Table 1. Complete Sequence of NR2F1-AS1
GTTTTGAGATCGCTCCCCTAGGCAGGCTCAGGCGGGCGCGGAGCCGCG<br>CGGAGTGACAAAGCCGCCGCTGCCGCCGCCGGGGGTGGGAGCCACGC<br>TCGCCCGCCCGCCCCTCGACTCGCGAGGGCGTAAAAGTTTGTCTCAGCT<br>CGAGCATTCCTAGCGCCAACACGCCCTCAGGTAAAGGTGGAAGTAAATG<br>GCCACGCTGTATTGACAGAGCAGGTAGATGAAACTCAAGAGAAAAGGTG<br>TGGATAAATGAAACTAGCCCATGATGAACCTGTTTTCTCCGTGACCACAAT<br>ATTAACCAGGATGAATGGCGGTGGCAGTGGGCCTGCATCACAGGTTGCA<br>GCAGATGTTCTCAATATTTCTATTAAAATTTCCTTATTTCCATATGCAAGAGG<br>AGCCCCAGAGCTGCATCCTTATGGTAGCTACCATGCCGTGATGTAAGCTG<br>CCATTCACTGGGGAGCTTTGGCAATAGAATTGGCTAGATCAGGAAGCCTA<br>TGTCAACTATGGAACAACAACTTGCAGCTCATCTCTATTGATGATGGAATC<br>TCGCTATGTCACCAGGCTGGAGTGCAGTGGCGCAACCTCGGCTCAGCG<br>CAACATCCGCCTCCCGGGTTCAAGTGATTCTCCTACCTCAGCCTCCTGAG<br>TAGCTGGGATTACAGGAATCTAATGACCATTGAACTGTGGCCATGACTTCA<br>ATTACAAGTATTTTCTTCACTAATGTCAGACTGTGGGCCTTTGTATATACAC<br>TTTTAACAAACTTTTTGGAAGTGCAATACATATCGCAACAACTTCTCAATGC<br>TGAATCATAGTATTGGAAGACATTTTAAGCAACAATTAAACTCAACTTATTTA<br>CTACCAATAATGTACATAAGTCAGCTGAAGTGAGGATAACATGAATAGCAC<br>AAATACATTATGATTGCTGCCTGGCCAACAGTAATTATAAGAAACTGTCAGT<br>TTTGAGATGTGTCTTGATTTCAGAGATGTTAAAAAAGTTTGTAAGTTACCAT<br>TGATAAAATATGAGTTTGCTTCCACAAATATTCTTTCTTCTGTCCTTCTGGA<br>ACTCAAGAGTAAACATATATTAGATTTTCTCACCATTTCATGCATGCTGGTT<br>CCTAGGCTACTCTCTGAGTCATTTTTTTTTTCTTATCTGCCCTCCAGTTCAC<br>TAGTTCTGTCATTTTCTGGGTCTGATCTGCTGCAAATGCAATACATTGAGT<br>TTTAAATTTCAGTCATTGTGTTTTCATTTCTAGAAATTTCATTTGACTCTTTC<br>TCAAATTACTTTTCCATTTTTTTTGTAGTTTCCTATCCCTGGAGATATTTTCA<br>ATCATGCCATTTAATTTTTTAAATACGCAAGTGTAGTTTTATAATTTGTGTCT<br>GATAATTCTAATATCTGAAGTCTTAGTAGGTCTGTTTCAGTTGTCCTGTAAT<br>TATGCATGTTTTTGCCCATGTTGCTTTGTTTCCTTGTATGCTTGGTTATCTT<br>TCTTACTGCCCTTGCAAAATTATTTGGAGAGTCCTGGAGGCTTAAATGAAG<br>ATAACTTTTAGGAATAATTTGCATTTGCTTTTGCCGAGGATCTTGGGATCAT<br>TTCAACCAAAATCTTAGCTTGAAATTCCGCAATCAATCAAGGTGATGAGAA<br>CCTGGTCAGTAAATCCTAATGAGGGCCAGGCTGTGGCCAAAGATTTTTAG<br>ACAGATGTTTCCCTCCTTTGCCATTTCTCTTTTCTTTCCTCCTCTGCTCCT<br>CTTAGTGTCCAGACAACCTTCATGGAAGTCCTTGGGGTGGGTGGGAAAT<br>GAGGACGGCAGGTTTCATTCTAGGTATAGCCCTTTGAGGTCTAACTTAATG<br>CAGAGAGAGTTTTCTGGTCTATGTGCCACCTTACATGGGATCTTGAGCCT<br>TGATTTCTAAACCCCTTGCCTTTGATGTTTCAGAATTGAAGCTTAAATTTGC<br>CAACAGGCAAATGTCCTCAAGGCAAATTGGCTTAGGTGTTCGATATTCTTC<br>TTACCAGAAAGGTAAACTTGGGTTTTTTACTTCACCCAGGATTTGGCATGA<br>CAGTTCCCCATTATCTTGTCAGCTTAGTGCTTTAAGTGATTGCATTTAAAAA<br>AATTCTGCATTTTCAGTTTTTAGCAGGAGGAATGGCCTCAACTAGTGGTTT<br>TCAACTGGGGATGATTTTTTTCCCCTAGGGGACATTTGATAATGTTTGGAG<br>ATGTTTTAGTTGTTAGAACTGGGGTTGGGGGAACTACTGTCATCTGGTGG<br>GGAGAGACCGGCAATGCTGTTGATCTTACTATAATGCACAGGCCACCCCC<br>AACAACAAACAAATTACATAGCCCCAAATATCAATAATGCCAAGGTCCAAAT<br>AACCTAACCTTCCATTACTGAAAATAGAAGTTCAAGAAGATAGTTTATAATTT<br>AAAAATTTTTAGGTCTTAATAAGTTGTCATACTTTCACAGGTTTTATTTCATA<br>CTAAGTAATTTATTTCAAATTTTACATTCCAATTACAGAAAATTTTCTAAGCC<br>AGTTTTTGTTCTTCATGAGTATTACTATCAAGGTCACATTTTTCATCTAGTAT

GTTTTACCCTGGTACTTGTTGGCAGGGTCAGGACTAAGGTGAGATGCGC
AAGGCATTTGCCTCACACAAAATGCACAATGAATAAAATGTCAAAACTTTA
AATAAAGACAGAATCACTAAAAGTACTTTGCCATATTGGAACCTGAGGCAA
AAAGAAAAATAAGTAATAATATTTAAAATTTTGATCTCTTGTTCATTGTAGATT
TTTACATTAATTTTGATTGTTTAAAACATTGCATTAAAATATTATTTAATGTGT
TTGCTGAGTTTTTTTTTGACACCCCCTTAAATTTGCACCAATGTGAGTGTC
TTGCTTTCCTTATTTGATTCCTGCCCTGCTTGTTATTGAGTCAGTCTGGTT
GGGTAGCTCATACAGGATGGAGAGGCCAGGGCTGACAATAG

Supplementary Table 2. NAS1-interacting candidate proteins identified by RNA pull down-Mass Spec analysis

	Swiss-Prot ID	Gene Symbol	Description	PSMs of differential SDS-PAGE bands		Mass Spec intensities of whole elution						
				Anti-sense	Sense	Anti-sense repeat 1	Anti-sense repeat 2	Anti-sense repeat 3	Sense repeat 1	Sense repeat 2	Sense repeat 3	Fold Change
1	P26599	PTBP1	Polypyrimidine tract-binding protein 1	14	112	4.93E7	2.21E7	3.67E7	2.90E8	3.23E8	2.11E8	7.63
2	P52272-2	HNRNPM	Isoform 2 of Heterogeneous nuclear ribonucleoprotein M	20	95	1.31E8	1.29E8	1.91E8	5.81E8	4.73E8	3.91E8	3.20
3	B4DLW8	DDX5	Probable ATP-dependent RNA helicase DDX5	11	58	5.20E7	3.69E7	6.79E7	1.56E8	1.23E8	1.05E8	2.45
4	O95758-1	PTBP3	Isoform 1 of Polypyrimidine tract-binding protein 3	7	15	8.52E6	6.39E6	6.45E6	6.77E7	4.34E7	3.18E7	6.69
5	F5GZS0	DHX36	ATP-dependent RNA helicase DHX36	0	8	5.87E7	4.45E7	7.43E7	2.36E8	2.15E8	1.51E8	3.39
6	P26368-2	U2AF2	Isoform 2 of Splicing factor U2AF 65 kDa subunit	0	6	1.89E7	1.39E7	0.00E0	1.26E8	1.29E8	8.68E7	10.43

PSMs: peptides-spectrum matches

Supplementary Table 3. Sequence of NR2F1 5'UTR segements	
**5' UTR (1-1802 bp)**	GGAAACATTGCTCTGCGAGTCCTCCCGTTTAAACGCATTCAATTTTTGGGT<br>CTCTCTCTCTCTCTCTCTCTCTCTTCTTCTCTTCTCTCTCTCTCTCTCTCC<br>TCTCTCTCTCTCCTCTTTCTCCCCCCTCTCTCCCTCCTCTCCTCTCTCTCTC<br>CTCTCTTCTCTGAACCTCTCTTCTCTTTCTCTTTTCTTACATATTCTACTAGT<br>TGTTTTCCCCTTCTTCTTCTCCTTTCTCTCCTTTTTCTCCCTCCTCCTTGTCT<br>CTTTTACTCCATTCCTGCAGAAGAGAGAGGGCTAAACTTAAAGAAAAAAA<br>AAAGGAGGAGGAGGAGGAGGAGGCACCCCCTTCGTATTCTTCTTATCGTT<br>ATTTTATACATATATGATTTTTTTTGGAGGGAGGGTGTTGGTTGCCGGCTGA<br>AGAGCACTTATTTAAAATACTAAAAAAAGAACATTTTTGGGCGATCTCCAG<br>GGTTTTTTTAACTAGCTCTGTGTGTTATAGCAGAAGAAGCAGAAGAAGGA<br>GCAAGAAAGAGGAAAAGAAGAGGATTATTTATTCGACCTACTTTGGATGT<br>CTCTCTCGCTTTTCCTTTTTCCTTTTTTTGGCAATTATTTTCTTCTGATTTTTA<br>TTTTTTCTATTTCGCTGTGATTTCGTCGCCGGCGTGAATTATCCCGTATTTT<br>TCTCCCCCTTCCGTCACCTCCCGAAAGAAGAAGGCAGCGAGAGCCCGG<br>CGCCACCGGCACAACAAAAAGAGCAAAGTGTGTGATCTTCCTCGCCGGC<br>TGCCTCCCGCTCTCCAGCGCTGCCTTCCTGAATGGCTGGCTGCGTCCGG<br>CCCTGGACCTGGCCCCCCGACACCCGCGCGCCCTGATCGCCGGCGGCA<br>GCCTCGCCCAGCGCCCTGCTCGGCTCACCGCGCTCCCCGCACTCCCGA<br>GCCCGGCGAGGGCTCCCGCCGGGACAGCGGCGGCGCCGCGGGCGGCC<br>CCGGCCTCCGCTCGCGCTCCGGCTGCGGCCCCGACTCCTGCTCGGACTC<br>CGGCCCGGGTCCCGGCTCCTCCAGCGGCGCTCGCCGCAGCAGCTCCGG<br>CGGCAGCTCCAGCGGCGCCTGCAGCCGCGACCTCCTCCTCCTCCGCCG<br>CCGCCGCCGCCTCCGCCCTCGCCGGCTTCCTCTATGTCGGCTCAGCCCG<br>CGCGCTGCGCGTAGCCCGAGCGGCCGGCGGGCGGGCGCCCGGCGCGG<br>GTGAGCGACTGTGTGTGCGAGTGTGTGTGTGCGCGGGGGTGCGGGCGA<br>GGCGGAGGGCGAGTGTGTGCGCGCGCCGTGGCCCATGCCCGCCGCCCC<br>CGGCGCTGCGCCCCGCGCCGCTCCCGGCTGCCGCCTGTGCCATTTCTGA<br>TTTTGCAACTTGGGGAAGAAGAAAAAAGCGAGAGAAGGGAGCTTGCTC<br>GCCGGGGGGTGGGGAGGGGGGAAGGAGAGCGCGGCCCCCCCAGGAAC<br>GGAGCGCGGGGGGAGCGGGCGAGGGGAGCAGGGGTGTTGGGGGGGG<br>AGCCTGAGAGCCTGGGGGGGCTGCAAAAAGAGAGAAAGAAAACAGCA<br>GGAACCACAACAAAACGCCAGCAGGGCGGGCGGGCGCGCAGCAGCAG<br>CGGGGCGGCCGAGGCAGTAGCGGCGGCAGCGGCGGCGGCGGCGGAGG<br>CAGCGGCCGGTGTCCGGCTCGGGCTCGGCTCCTGCGACCCCGGGGCGC<br>CCGGCGGGCCCCCCGCCCCCTCCCCCTCCCCCCTTCCCCTTCCCCTTCC<br>CCTCCCAGCGCGCCCGCGCGCCCCGCGGCCCTCGGCGAGCAGCTCGGC<br>TCCCCCCAGCGCTCCCCGGGCCCAAAGAT
**5'UTR-PT (1-800 bp)**	GGAAACATTGCTCTGCGAGTCCTCCCGTTTAAACGCATTCAATTTTTGGGT<br>CTCTCTCTCTCTCTCTCTCTCTCTTCTTCTCTTCTCTCTCTCTCTCTCTCC<br>TCTCTCTCTCTCCTCTTTCTCCCCCCTCTCTCCCTCCTCTCCTCTCTCTCTC<br>CTCTCTTCTCTGAACCTCTCTTCTCTTTCTCTTTTCTTACATATTCTACTAGT<br>TGTTTTCCCCTTCTTCTTCTCCTTTCTCTCCTTTTTCTCCCTCCTCCTTGTCT<br>CTTTTACTCCATTCCTGCAGAAGAGAGAGGGCTAAACTTAAAGAAAAAAA<br>AAAGGAGGAGGAGGAGGAGGAGGCACCCCCTTCGTATTCTTCTTATCGTT

	ATTTTATACATATATGATTTTTTTTGGAGGGAGGGTGTTGGTTGCCGGCTGA<br>AGAGCACTTATTTAAAATACTAAAAAAAGAACATTTTTGGGCGATCTCCAG<br>GGTTTTTTTAACTAGCTCTGTGTGTTATAGCAGAAGAAGCAGAAGAAGGA<br>GCAAGAAAGAGGAAAAGAAGAGGATTATTTATTCGACCTACTTTGGATGT<br>CTCTCTCGCTTTTCCTTTTTCCTTTTTTTGGCAATTATTTTCTTCTGATTTTTA<br>TTTTTTCTATTTCGCTGTGATTTCGTCGCCGGCGTGAATTATCCCGTATTTT<br>TCTCCCCCTTCCGTCACCTCCCGAAAGAAGAAGGCAGCGAGAGCCCGG<br>CGCCACCGGCACAACAAAAAGAGCAAAGTGTGTGATCTTCCTCGCCGGC<br>TGCCTCCCGCTCTCCAGCGCTGCCTTCCTGAA
**5'UTR-GC (781-1802 bp)**	TCCAGCGCTGCCTTCCTGAATGGCTGGCTGCGTCCGGCCCTGGACCTGG<br>CCCCCCGACACCCGCGCGCCCTGATCGCCGGCGGCAGCCTCGCCCAGC<br>GCCCTGCTCGGCTCACCGCGCTCCCCGCACTCCCGAGCCCGGCGAGGG<br>CTCCCGCCGGGACAGCGGCGGCGCCGCGGGCGGCCCCGGCCTCCGCT<br>CGCGCTCCGGCTGCGGCCCCGACTCCTGCTCGGACTCCGGCCCGGGTC<br>CCGGCTCCTCCAGCGGCGCTCGCCGCAGCAGCTCCGGCGGCAGCTCCA<br>GCGGCGCCTGCAGCCGCGACCTCCTCCTCCTCCGCCGCCGCCGCCGCC<br>TCCGCCCTCGCCGGCTTCCTCTATGTCGGCTCAGCCCGCGCGCTGCGCG<br>TAGCCCGAGCGGCCGGCGGGCGGGCGCCCGGCGCGGGTGAGCGACTG<br>TGTGTGCGAGTGTGTGTGTGCGCGGGGGTGCGGGCGAGGCGGAGGGCG<br>AGTGTGTGCGCGCGCCGTGGCCCATGCCCGCCGCCCCCGGCGCTGCGC<br>CCCGCGCCGCTCCCGGCTGCCGCCTGTGCCATTTCTGATTTTGCAACTTG<br>GGGAAGAAGAAAAAAAGCGAGAGAAGGGAGCTTGCTCGCCGGGGGGTG<br>GGGAGGGGGGAAGGAGAGCGCGGCCCCCCCAGGAACGGAGCGCGGG<br>GGGAGCGGGCGAGGGGAGCAGGGGTGTTGGGGGGGGAGCCTGAGAGC<br>CTGGGGGGGCTGCAAAAAGAGAGAAAGAAAACAGCAGGAACCACAACA<br>AAACGCCAGCAGGGCGGGCGGGCGCGCAGCAGCAGCGGGGCGGCCGA<br>GGCAGTAGCGGCGGCAGCGGCGGCGGCGGCGGAGGCAGCGGCCGGTG<br>TCCGGCTCGGGCTCGGCTCCTGCGACCCCGGGGCGCCCGGCGGGCCCC<br>CCGCCCCCTCCCCCTCCCCCCTTCCCCTTCCCCTTCCCCTCCCAGCGCG<br>CCCGCGCGCCCCGCGGCCCTCGGCGAGCAGCTCGGCTCCCCCCAGCGC<br>TCCCCGGGCCCAAAGAT

**Supplementary Table 4. Gene sets used for GSEA and ssGSEA analyses**	
NOTE: E-CSC_signature and M-CSC_signature in ssGSEA analysis are the combination of UP and DOWN gene sets, respectively.	
Names of Gene sets	Genes
NAS1_KD_signature (FC>1.5 by NAS1 KD in MCF10CA1h)	KM-PA-2,SLC2A3P1,F8A2,TMEFF1,AC004889,AC009133,ASB3,DYX1C1-CCPG1,AC104534,F11R,AP000275,DHRS4-AS1,RP11-512M8,MUC2,RP11-173P15,RPL21P119,AC008738,RPP14,RP11-407N17,RP11-504P24,MMEL1,ZNF658B,MIR205HG,ARHGAP19-SLIT1,S100A14,TREM2,BMF,IRF6,FAT2,NGFR,ANXA8L1,CAMK2B,ANXA8,TP73,KB-1572G7,FOXI1,IMPDH1P10,DNAJC25-GNG10,PA2G4P4,RP11-265D17,GRTP1,PVRIG2P,HNRNPA1P33,SLAIN1,THSD7A,CTD-2192J16,SYT8,SFRP1,OR2A1-AS1,COL17A1,RP11-469H8,KRT17,WFDC2,LPHN3,HMGCS2,NCALD,MDFI,RP11-498C9,FGF1,LZTS1,HIST2H4A,RNASEK-C17orf49,KRT6B,RP11-392E22,KRT86,TMEM40,UGT1A6,AC004466,RP11-439A17,RND2,RP11-867G23,C3orf67,PCOLCE,KCNAB3,RP11-571M6,CAPN6,XDH,ARHGAP25,CTC-251D13,ANXA8L2,GPR56,ANKRD22,AL353354,CXCR4,AL603965,RP11-77H9,RYR3,ADRA2B,POC1B-GALNT4,ZNF204P,ARTN,C11orf35,SMOC1,PTAFR,NUTM2E,RP11-651P23,PCSK5,ISG20,DHRS13,LMTK3,MSTO2P,TBC1D3G,HLA-DPB1,RP11-148K1,GPRC5B,PLA2G4B,SOSTDC1,FAM212A,CTD-3232M19,PALMD,TNFRSF19,SYCE1L,RP11-981G7,SUSD4,CCAT1,PALM2-AKAP2,UNC5C,SLC16A14,FREM2,RP11-574K11,ARID5A,RGMA,TRIM29,MYO1D,FAM83A,GUCY1A3,VSNL1,TINAGL1,LSR,KRT81,LRP4,RP4-669L17,RP11-798M19,HAS2-AS1,SNX29P2,MACC1,ZC4H2,C1orf116,PKNOX2,SFT2D3,RHOV,BDKRB2,MYLK2,POLR2J2,RASSF6,ARHGAP28,PAQR8,GRIP1,MUC5AC,RP11-154H23,TNNT1,RP11-141O15,RP11-106M3,AC022007,AACSP1,GPC2,RP11-262H14,PAK6,ABCA10,TGM4,HNRNPA1P49,AC007276,NAA60,CALB2,SERPINF2,KRT15,RP11-77P6,HAPLN1,PIK3C2G,SPTB,RP11-599B13,RP1-228P16,RP11-205M20,MUC5B,RP11-24N18,PCDHGA12,RP11-422J8,DLL1,CDH11,NPM1P24,EDIL3,GRB7,RPL21P120,SLC5A3,RP11-153M3,C17orf104,FA2H,FUT1,RNF144B,RP11-339B21,KRT23,RP11-298I3,RANP1,CTD-3092A11,CHKB-CPT1B,LBH,RP13-516M14,LINC00265,NUTM2G,NICN1-AS1,GS1-124K5,RP5-882C2,F5,AC009404,GNAO1,FXYD3,MBLAC1,RP11-34P13,FGFR4,TNS4,RP11-34P13,RP4-559A3,TMEM92,PRSS53,NFATC4,GPR75,HIST2H3PS2,KIAA1875,CDH1,ABHD14A-ACY1,LRAT,CTA-384D8,DUSP8P5,PLCH2,HSPE1-MOB4,AC018766,RP11-121L10,FAM184A,RP11-345P4,AL049840,CHRM3,RP13-582O9,MACROD1,MYEOV,LA16c-60H5,NNAT,UNC5B,PCYT1B,RP11-104G3,ERVMER34-1,KCNJ14,CEMP1,DAAM2,USP2-AS1,CD82,CHRM3-AS2,RP11-73M18,SLC4A11,AL136419,RP11-548H3,PBX4,RP4-673M15,RP11-111F5,SLC2A9,RP11-273G15,ZNF385A,THAP8,C15orf37,NES,IGFLR1,FAM211A,HES6,CTC-462L7,ZNF821,LIME1,C18orf56,CAPS,RP11-876N24,SCNN1B,SERPINF1,BCL11B,RP11-849H4,HOXA6,FRMD4B,PVRL1,CHRNA10,RP11-6N17,FCRLB,ODF3B,PCDHGB6,N

	ANOS1,FAM27B,CTD-2545M3,NFATC1,NUTM2A,RPSAP54,NEIL1,B3GNT3,CTRL,ARHGAP33,AP001007,BDH1,PMS2P4,AC002398,FZD8,NAP1L4P1,RP5-827C21,FAM151B,S100A2,TMEM238,C1orf172,RP11-152F13,MYB,RPS6KL1,TMEM158,DCHS1,EIF4A1P2,LINC00473,ST14,ADRA1B,HIST2H2BC,HCN3,WNT10B,RP11-178G16,EXPH5,RDH16,RP11-65J3,CDS1,EGR3,PRSS12,CSPG4P8,RP11-810M2,ASMTL-AS1,DEF6,PMF1-BGLAP,MICAL2,ARHGAP8,SDC1,TUBBP1,KIAA1614,CCDC74A,C2orf27A,RP11-16P6,SFN,STAC,SERBP1P1,PROB1,CTD-2267D19,CARD10,HOOK1,CASZ1,C1orf233,SLC7A5P1,CA2,NUP62CL,SLC27A3,JAG2,AGER,IRX1,LRRC45,RP11-244K5,KHK,MSH5,RP11-723O4,MAP2K6,IRX2,CTSV,CIDEB,DNAAF3,LINC00623,RP11-464F9,SHISA2,KRT6A,B3GNT4,RCOR2,CHTF18,MFNG,IGSF9,ZHX1-C8ORF76,ISL2,HR,CEBPB,SVILP1,PPP1R1B,LRRC26,RP11-1006G14,C4orf48,ZNF165,SLX1A-SULT1A3,PI4KAP1,NDRG2,GPR3,SH3RF2,RP11-556K13,RP11-650L12,SRRM5,PEX5L,NHLH2,SPAG4,CKLF-CMTM1,CTD-2517M14,MROH6,C14orf169,RP11-1246C19,PRELID2,EGFL8,OPLAH,TFCP2L1,RP11-313P13,HOXA-AS2,RP11-228B15,EFNA1,SULF2,CDH3,RP11-478C19,STAB2,LINC01001,PLAC9,SRGAP3,BMP8B,FAM27A,TSHZ2,AC103801,AMH,FSCN1,CCDC65,SERINC2,SGPP2,SLC37A2,ANKRD9,SAPCD2,SETBP1,F12,PRSS16,SPINT1,RP11-468E2,ETV2,DNM3,WNK2,AC137932,TRIM7,RP3-512B11,AC016773,RP11-752G15,GK,JPH1,ZNRF2P2,RP11-274B21,AC034220,ASIC3,RP11-496I2,ZGLP1,MISP,DSC3,PKN3,RP11-793H13,AC007318,ISL1,RTEL1-TNFRSF6B,COL1A2,HES7,RP5-998N21,GRK6,MYBPC1,PALM2,HS3ST3B1,CSRP2,EVL,COL9A3,RP11-545E17,RP11-169K16,PPP1R13L,SLC45A3,GPC4,CD274,KREMEN1,ADM5,C5orf46,ZNF34,_SEPT4,HAS3,GHDC,TMCC2,RPL5P1,SOGA2,SCNN1G,RNF152,PPP2R3B,LRRC37B,DDX12P,GLS2,PAX6,CCDC120,MTFP1,IGSF3,RP11-195F19,IRX4,GLIS1,CHDH,CD96,DPYSL3,LSP1,RP11-256P1,ZNF620,AC000089,FAM203A,RP11-706O15,IGFL4,LA16c-358B7,ARL17B,HOXA11-AS,NKAPP1,CLEC11A,C15orf38,RP11-345J4,OSR1,NRARP,KIAA0040,TSTD1,AP3B2,KCNK5,ARHGEF4,WDFY3-AS2,CDT1,ISM1,HOXC5,TPRXL,GJB3,PPP1R14C,RGS20,CCDC74B,UBE2SP1,CSPG4,TMEM106A,MARVELD2,SLX1B,FANCE,FGFBP1,RP11-872D17,CTB-58E17,RIMS1,RP11-282O18,MILR1,RP11-294J22,TMSB4XP8,CCBL1,UBXN11,EPHB3,LDHAP4,HINT2,EYA1,RP11-442A13,CDC42BPG,RP11-37C7,DKK3,HOXA-AS4,LURAP1,SYK,MAMDC4,FUT5,TOR4A,BCL2L11,GYLTL1B,INPP5A,GCSAM,RP1-241P17,PAQR6,COMMD3-BMI1,SLC37A4,JMJD7,RP11-73M18,PKMYT1,LRRC56,PART1,AFF3,BMS1P10,RP11-423P10,FAM78B,TMC7,SNHG10,STMN3,ACOX2,SLC29A1,UNC13A,SRSF8,AC073072,C5orf38,CSPG4P9,SNORD109A,SOCS1,ARG2,ATP8A1,HMGB1P1,INPP5J,CASKIN2,RPS26P47,NRM,MEST,ZNF213,GPER1,HAUS6P1,THOC6,NSUN5,KCNRG,RP11-72I8,ZNF488,POMZP3,RP11-115C21,RP11-726G1,C10orf95,RP11-589M4,B4GALNT1,C9orf117,ZNF316,AC084219,RP11-698N11,PLCB1,HECW2,RP11-392E22,HES4,PLA2G

	3,A1BG,SULT1E1,CMTM1,RP11-206L10,DCPS,PKP3,PCLO,MPP2,CHKA,LINC00857,ARHGEF16,TPI1P1,KCNC4,MESDC1,RASA4B,NUDT8,CSPG4P10,NCAM1,FOXC2,RP11-111F5,RP11-31F15,DOK3,MED24,AL590822,RP11-395P17
NR2F1OE_signature (FC>1.5 by NR2F1 OE in MCF10CA1h)	POLR2J2,AP000275,C10orf32-ASMT,RP11-566K11,CXCR4,MRPL30,RP11-571M6,SCNN1B,SOHLH2,NR2F1,INHBE,DNAJC25-GNG10,RP11-73M18,SCRG1,APCDD1L,RP11-746M1,CHAC1,SDHAP2,MIA-RAB4B,FUT1,APCDD1L-AS1,HYAL1,ITGB2-AS1,UNC13A,WNK4,RP11-834C11,GPR55,SPINK13,TLR5,GP1BB,ACAP1,IP6K3,S1PR1,DUSP5P1,TOLLIP-AS1,AC002116,ADM2,TBC1D3H,ABAT,ATAD3C,CYP1A1,ACVR1C,RASGEF1B,PCDHGA2,RP11-875O11,LAPTM5,CD96,AC120194,HIST2H3A,CCDC64,SCNN1G,RP11-422J8,SUSD4,SYCE1L,RP11-38M8,LSR,SULT1A2,RP11-50D9,SLC1A4,F8A3,TREX2,LY75-CD302,CYP4F11,CLIC3,MYL9,CGNL1,ALDH1L2,C3orf80,AL035252,PIP5KL1,RP11-723O4,PLA2G4B,RP11-983P16,LINGO2,HNRNPA1P4,CAMP,ASNS,NCAM1,CNIH3,RP11-805J14,FOSB,ADAP1,ALOXE3,RBM26-AS1,SLC7A11,RP11-723O4,RP11-1109F11,TRPV1,ANKRD24,PART1,JAKMIP2,SPP1,SNAI3,LINC00473,RP11-592B15,FAM132B,KDR,DDIT3,TCF7,RP11-726G1,KLF4,ADORA2A,BX088651,LTB4R2,RP11-263K4,CTD-2555C10,ITGB2,DPT,IL21R,RP11-412D9,DDR2,AC005355,AC009133,SLC6A12,LINC00669,FGFBP1,C9orf169,TP53AIP1,RP11-111F5,ZNF287,CHMP4C,VAMP5,COL1A2,TSSK6,RP11-727F15,ARG2,SERF1A,DNER,MYRF,TRPM3,PIK3AP1,MKLN1-AS2,GPT2,C15ORF37,CTA-204B4,CD226,XXbac-B562F10,STC2,CD52,PTK6,ZNF583,HMGN3-AS1,RIBC1,RP4-569M23,RPL5P23,SOCS2-AS1,RP11-1149O23,KBTBD11,KB-1572G7,KRT16,SH2D5,REEP6,PLA2G3,AC007255,EEF1A1P12,HLX,PDZD7,PCK2,EGR2,LAMP3,WNT2B,CRYGS,MFSD4,RBM20,AC138035,GPR162,AC092811,PID1,FREM2,ZFP69B,PTGS1,RP11-119F19,SHF,RP13-895J2,INPP5D,RGS16,PDGFRA,NOS1AP,BMP7,TRIB3,OR2A9P,GAMT,DND1,ZNF606,DAPK1,EGR1,ACTL8,AC112721,LRRC37B,ATP8B1,BMS1P11,SLC16A13,RP11-326K13,RP11-98J23,TOB2P1,AL139385,VAX2,SULT1E1,C20orf196,GLI1,AZGP1,DNAAF3,CSPG4,SLC43A1,SMOC1,IL1RL2,FMO5,GPR160,KREMEN2,GPR124,CPT1C,CALHM3,ZMYM6,LCN10,CTB-52I2,SLC7A5,RNF32,TNXB,FAM225B,RP11-165J3,C17orf67,PIK3CD,CLCN5,PTPRO,GOLGA8Q,ERVMER34-1,RP11-342M1,ARNT2,CAPN3,ZNF821,RSC1A1,CTD-2369P2,GDF15,PSAT1,EFCAB13,CTD-2008A1,TNS4,DHRS13,SWSAP1,MUC6,FYN,RP11-13A1,B3GNT4,SLC27A2,ZRSR1,SMPD3,SYTL3,GPC4,LRRC17,ECM2,P2RX5-TAX1BP3,TNFSF15,RAMP1,FAM83A,PTHLH,IFRD1,LINC00511,C9orf117,AC012318,RP11-434C1,OSER1-AS1,SOCS2,MYO7A,KCNMB4,SESN2,PLCB2,PCDHGA10,NBL1,TNFAIP6,CERS1,VIM-AS1,RP11-163E9,RARRES1,RP11-96C23,MAFF,TAPT1-AS1,DUSP5,AC007743,APOE,RP11-392E22,RP1-179N16,RBM14-RBM4,RP11-31F15,AC125232,BDNF,PITRM1-AS1,TNNT1,FAM72A,RTN4R,RP11-416I2,ANKRD1,TBC1D3C,RASAL1,ZNF701,VGF,MAL2,GOLGA2B,C14orf182,YBX1

	P10,RP11-529K1,RPL4P5,RP11-24N18,CD14,NPPA-AS1,POC1B-GALNT4,IL8,OGDHL,GRAMD1B,AKNAD1,RP11-48O20,ENO1-IT1,AQP5,ENOX1,RP11-434D9,GSTT2B,ABI3BP,ADAMTSL1,LINC00936,RP11-298J20,AC022007,NRG1,CCAT1,MATN1-AS1,BGN,CRYAB,ROCK1P1,SP4,SEPT3,CCL20,CATSPER2P1,CD68,VAC14-AS1,RP5-1136G13,RP3-324O17,CX3CL1,SPINT1,SPATA6L,HNMT,PSPH,TUFT1,RP11-96H19,RASSF4,UNC13D,INPP5J,AMOT,SPDYE2,RLTPR,GPSM3,KRT5,BRSK1,RELL2,GGN,RP11-316M1,LINC00313,HAPLN3,AC006449,KCNT1,RP11-262H14,AC011933,PCDHGB7,MLANA,AC018766,LEAP2,EPCAM,SLC3A2,FAM66C,HIST1H3H,AC091729,GSG1,RP11-181G12,AC005786,RHBDL2,PDE7B,C5,FAM27E3,DSG3,EEF1DP2,SUSD2,NTNG2,RN7SK,RP11-228B15,HSPB8,PITX2,RABGEF1,RP11-1415C14,MIR146A,SLC45A1,SFT2D3,LRRC24,PPP1R14C,AL358781,RP11-268J15,NPIPA7,RP5-1086K13,PXK,MAP3K7CL,AC144652,ASAH2,ERG
ΔNp63_UP (FC >3 by ΔNp63 in MCF7)	KRTAP5-9,ANXA1,RPL41,CEL,NTN4,GPNMB,CAV1,BCL2A1,EGFL6,SNTN,ACP5,STK3,SLC37A2,C2orf42,C2orf84,WBSCR28,UGT1A8,TREH,NT5E,TP63,FAM183B,A2ML1,GJB5,TMBIM1,C1orf88,LDB3,CD36,ANXA8L1,SPARCL1,SERPINF1,TRIM54,C2orf67,THEG,LY6G6D,COL28A1,DCAMKL1,ANKRD22,PSG8,GPR87,HCRTR2,CDC42EP3,MYOT,MARK1,ZNF61,GCA,KRT32,NPAS3,SPRN,SNAI2,OXGR1,CRYAB,VTCN1,MFSD2,CPXM2,PRKG2,WNT5B,KLHDC1,ACVR1C,KIAA43,C3orf18,FGF1,C6orf17,CCDC3,MGAT4C,C1orf161,AQP3,HAP1,ILVBL,ATPGD1,BTG2,S1A8,DQX1,GDPD2,GPC3,TMEM4,FOS,CHRNA3,LAMB3,CYP1A1,ETV7,MPZL2,FAM183A,DGKA,SNCG,GHR,DKK1,YPEL2,C1orf16,ID3,DYNC1I1,C1orf11,TMPRSS7,FBXO32,BLNK,CYP4B1,GLS2,FILIP1L,MMP25,BCL2L1,EPHA4,PDE4DIP,TLE4,ACMSD,FABP6,FEZ1,PCP4L1,PBXIP1,SRGAP3,GGT6,TMEM117,CLCA2,ASB9,PNRC1,MAF,PROCR,RASIP1,KRT15,TP53INP1,BOC,HBD,ATP6V1C2,CDKN1A,C17orf13,KISS1,SPATA18,PIK3IP1,SLC22A4,PAPSS2,SCARA3,COL4A5,SESN1,PHYH,ST6GAL1,SOCS2,SLC31A2,ENPP2,CYFIP2,SERPINB5,SPINK4,MCC,PADI3,LAMB1,PDK4,CPE,DYRK3,PIK3R3,KLHL24,RALGPS1,ID4,SNCAIP,C5orf41,SART2,RRAGD,OPTN,KIAA135,IL25,C17orf91,C3orf54,STOX1,RUNDC3B,EPHX2,PSTPIP2,SLC27A2,AHNAK2,PLAU,LGI2,C18orf1,ZDHHC8P,GPX2,JAG1,KLF8,RDX,UNC5C,ELOVL4,ARHGAP24,SESN3,TMCC3,DHRS3,RUNDC3A,BBOX1,FGFR3,MXD4,GABARAPL1,ST3GAL5,MATN2,FAM46A,INPP1,MEIS1,HIST1H4H,C14orf147,TMEM159,KIF17,MPP2,COL16A1,SYTL1,WISP2,YPEL5,ID1,GDPD1,CCDC154,MRAS,FBLN7,LMO2,MVP,KAT2B,MR1,COL7A1,XG,NDRG4,GADD45A,PRNP,GPR115,TMC4,FAM49A,SLC16A4,PDLIM1,DLX2,FAM83B,KIAA137,PTPRQ,SRPX,MBNL2,ENPEP,PPL,SMARCD3,PSG2,PARP12,LDLRAD1,CDH15,TMEM17,DIO1,C9orf61,SLC46A3,C1QTNF3,NEB,CLYBL,TSGA2,NLRP1,TTC28,GTF2IRD2,TXNDC6,ZNF488,SLC44A3,BEX2,C17orf44,TCP11L2,DEF6,PTGS1,USP31,APCDD1,ITGB7,KIAA1984,ABCC5,FAM134B,TLR8,FADS3,CCNG2,PLCG2,NIPSNAP3B,AKR1C2,CAV2,DHRS8,FHIT,ALDH5A1,EFNA1,TRPV2,GRN,TSGA1,USP2,HTRA1,

	PLEKHB1,RASSF4,ABCA5,RAB4B,FOXO4,ANXA2,PERP,CTNND2,ORAI3,GTF2A1L,AMOT,QPCT,GPLD1,SVOPL,ANTXR2,KIFC3,ANKRD42,EFEMP1,SLC3A3,S1A2,DBP,CD59,ELAC1,JARID1B,SLC29A3,FHL2,LIMA1,ADORA2B,PDCD4,CNNM2,C4orf18,PHLDB2,CROT,SC5DL,ITGB4,LCA5,MYOF,BEX4,CD82,C2orf18,FGFR2,SLC2A11,TAX1BP3,SERPINA3,SH3YL1,GARNL3,C15orf52,DDIT4,ZNF345,SUPT3H,BCAS3,ALDH7A1,TTLL3,SESN2,CDK6,RAB7L1,LRRK1,PLEKHG6,GLRA4,YPEL1,C1orf168,TNFRSF12A,GSTA4,HOXC13,FOLR1,MTSS1,PDLIM7,FXYD3,RDH16,SLC5A12,C19orf66,TPK1,CTSH,ZNF65,SDC1,FMO4,ARL4P,ZG16,C21orf55,C17orf55,BTN2A2,ANKRD56,FZD5,F2R,RBL2,EVPL,ZMAT3,SCD5,FOXO1A,MCOLN3,FUCA1,CDKL2,C9orf155,OLFML2A,EGFL9,ITPR1,ALS2CR4,LAPTM5,NR3C1,IRF1,GRIP1,PSMB1,AKD1,CCDC148,NPL,MSGN1,C9orf95,ZNF774,MUC8,TACSTD2,NUAK1,HIST2H2AA4,VAMP5,C6orf163,NUPL2,PTHLH,PLEKHG1,AHSA2,TF,C15orf33,SHC2,CRIP1,KCNA5,BTBD11,LYRM5,COL11A2,KIFAP3,ABCG1,ECHDC2,MAPK1,FAM89A,PGAP1,ERAP1,C3orf59,MYBPH,CAT,SEMA4A,PCDHB18,HDC,C7orf38,ANKRA2,HSPA4L,CYP4X1,FBRSL1,NPAL2,REP15,CRIM1,TNRC6B,DIAPH2,AP552.1,EXOC6B,PTGDR,NADSYN1,ERO1LB,LNX1,CALCA,CST3,PROS1,ANXA13,BDKRB2,LEPR,NBEA,DUSP14,DEGS1,OSR2,TEKT3,RGL1,HIST1H2AC
ΔNp63_DOWN (FC <1/3 by ΔNp63 in MCF7)	POLQ,NMU,C1orf162,ASCL3,PTGER2,APOC3,C8orf31,LEP,TFPI2,ANKRD3B,CXorf65,WDR62,CAPN13,OR4E2,C5orf56,C9orf1,C2orf175,C3,TMEM194B,CFC1,IFI27,NCBP2L,ZMYND1,SNAI1,SFRP1,DIO3,CXCR4,MCM1,CLSPN,FFAR2,DSCC1,FAM5B,FAM111B,CCNE2,RRM2,CENPF,RAD51,PLEKHK1,CDC45L,MND1,RAD51AP1,MKI67,EME1,SPC25,FAM64A,CCNA2,ERCC6L,KIF2C,SHCBP1,C14orf145,TRIP13,CDCA5,CDCA8,RAD54L,KIFC1,CDC2,KIF15,TOP2A,C15orf42,TK1,DLK1,OIP5,ESCO2,E2F2,BUB1,GINS2,SGOL1,MLF1IP,UHRF1,PLK1,GTSE1,LOXL3,MGP,ASF1B,CEP55,KIAA1524,CDC6,RHOH,NCAPG2,TROAP,MELK,MAD2L1,CENPE,C13orf3,TTK,C5orf34,HRASLS2,FAM54A,NCAPG,RIBC2,MYBL1,NCAPH,FAM83D,KRTAP5-9,PRIM1,GINS4,HCAP-G,MYBL2,PBK,KIF23,ORC6L,ATAD2,HMMR,CDCA3,BUB1B,HJURP,SMAD3,DIAPH3,AURKB,CCNB2,KNTC2,PRR11,BRCA1,SPDEF,FOXM1,CENPI,C6orf141,SLC4A1,TCF19,TMPRSS3,UBE2C,PSMC3IP,ANLN,C1orf112,TPX2,ZWINT,NEIL3,MCM2,KIF11,TYMS,KIF14,LIG1,CDKN3,DTL,VGF,NUSAP1,RACGAP1,BRCA2,FANCI,MASTL,FEN1,IQGAP3,ZNF367,GPR19,C6orf173,DEPDC1,CDC25C,SUSD3,UBE2T,DOK7,PLK4,FANCB,KIAA1324,BAMBI,GS1-484O17.2,NUF2,DLGAP5,CDCA2,MCM7,ARHGAP11A,PKMYT1,CENPA,CHAF1B,HSD17B6,PRC1,NEK2,ASPM,CENPM,GAL,BIRC5,WDHD1,CSTA,CHEK1,C6orf15,HIST1H1D,UCHL5IP,RNASEH2A,PHF19,CKS1B,POLA2,MCM5,C2orf172,RBL1,LMCD1,GGH,DEPDC1B,SCD,APOA1,FBLN2,CA2,KIF18A,LRP8,TMSL8,C11orf82,TUBA1B,SGOL2,C2orf127,KIF2B,MT2A,PIP5KL1,KNTC1,XBP1,TFF1,C15orf23,BRIP1,CENPH,SMC2,TRAIP,BARD1,GAS2L3,MCM6,MCM3,PCNA,XRCC2,RFC3,ITFG3,KIAA11,RAD54B,CCDC99,RFC4,KIF2A,R

	DM1,B3GNT6,WDR51A,HMGB2,FBXO5,IER3,ELOVL2,TRIM14,NRM,DBF4,NUDT1,C18orf24,GINS3,CENPO,FIGNL1,TMPO,MCM8,LMNB1,C16orf75,HIST1H1B,RET,ECT2,ALDH3B2,HIST1H4G,MT1P2,C18orf54,CENPN,CENPQ,H1F,MT1H,WDR76,GMNN,TUBB,CCDC15,PSRC1,CD32,HIST1H4B,PHLDA2,FANCD2,CDCA7L,CDC7,ORC1L,HICE1,POLE2,MT1B,AURKA,FAM129A,LOC81691,GALNT14,GINS1,MGLL,NCAPD3,CCNB1,HELLS,KCTD12,MNS1,BMP4,CDKN2C,SIDT1,RAMP1,HK2,TRGV7,CENPK,S1PR3,ATAD5,STK32B,SPANXD,RECQL4,FHOD1,TIPIN,C6orf167,DOK1,AMIGO2,DMC1,MCM4,SPIN4,FANCA,FAM181A,PNKD,CENPL,GSG2,CKLF,AKAP5,DBNDD2,SYTL4,FAR2,EXO1,ASRGL1,CXCL12,STMN1,PPIL5,RHOB,MSMB,ZNF9,CRELD2,BRI3BP,ST8SIA6,MTHFD1,CKS2,DTYMK,EXOSC8,CTPS,LYPD6,CHRNA5,CKAP2L,HIST1H2AL,TAF5,CBX5,TEX14,C13orf34,TMEM21,PCDH19,OLFM1,CENPP,SERTAD4,TUBA3C,C5orf35,PLEKHA7,FN1,KIF12,CHAF1A,C9orf14,DCLRE1B,DNAJC9,CORO1A,UBE2S,CDT1,PRTFDC1,C2orf7,POLD1,DHFR,CCDC68,PIF1,AXL,GCNT1,TIMELESS,PTGER4,STK31,RERG,TFCP2,MICB,SDK2,NR2C2AP,FGF18,CDC42EP2,SMC4,BX255925.2,WDR34,ANKRD32,GPR39,RPL39L,TFF2,MAK16,GTPBP1,GJB2,C9orf84,AP2364.4,POLD3,FBLN1,PDSS1,TFAP4,GSTCD,TMEM194A,ZDHHC14,ACAT2,DHRS2,METTL1,LIN9,FZD4,RFC2,MDC1,CARHSP1,FANCG,SLC25A19,TP73,MYB,VRK1,E2F7,GFRA1,NRXN3,ZNF473,HGD,PKD1L2,C4orf21,POLA1,ITGB3BP,TUBG1,ZBTB2,CEP152,C12orf48,FHDC1,SLC8A2,SUV39H2,TMEM48,CMBL,GPSM2,RECQL,DDX39,HES6,MXD3,CDK2,SPAG5,DLG3,SLC6A14,PLA2G4C,RCCD1,AGR2,NUP155,NCAPH2,CCND3,HSPA2,AEN,SDF2L1,C2orf16,TUBA1C,PIGW,IER5,RBP1,LRRC59,TTF2,LBR,C17orf53,INCENP,PRIM2,SNX1,RAP2C,SLCO4A1,LCP1,AMBP,DHTKD1,THRB,BOP1,BLM,STIL,ITPK1,C1orf135,MMD,RFC5,ATL3,CHTF18,SYCE2,RAI14,EDN1,PYCR1,LRP4
EMT_signature (pan cancer EMT markers, PMID 26420858)	ADAM12,ADAMTS12,ADAMTS2,AEBP1,ANGPTL2,ANTXR1,AXL,BNC2,CALD1,CDH2,CMTM3,CNRIP1,COL10A1,COL1A1,COL1A2,COL3A1,COL5A1,COL5A2,COL6A1,COL6A2,COL6A3,COL8A1,DACT1,EMP3,FAP,FBN1,FN1,GPC6,GYPC,HTRA1,INHBA,ITGA11,LOXL2,LRRC15,MMP2,MSRB3,NAP1L3,NID2,OLFML2B,PCOLCE,PDGFRB,PMP22,POSTN,SPARC,SPOCK1,SULF1,SYT11,THBS2,VCAN,VIM
E-BCSC_UP (FC >4 in CD44-ALDH+ vs CD44-ALDH-, GSE52262)	HGF,SYNE2,XIST,ZBED6,TCEB1,SLFN5,UBASH3B,ARSB,MSI2,P2RX7,TPTEP1,C6orf52,ZNF652,ALS2CR11,HIVEP2,KIAA1324,ZDHHC20,MAVS,NDUFB7,ZNF394,C1orf61,ZNF827,MRPL30,SGMS2,CTA-384D8.35,C20orf196,ALPK1,DNAJB9,CASZ1,FLCN,ENO2,ABCC1,RFX3,IGF1R,SRD5A1,CSNK1A1,MORF4L2,SLC25A44,AGAP6,LTBR,CREBBP,ZNF107,SRD5A1,ZDHHC21,ARHGAP19,WLS,C10orf54,ALS2CL,GTF2E1,CPS1,STAT3,CSPP1,SHROOM3,MED28,NUDT3,GCC2,INO80D,GCC1,KREMEN1,VAPB,TXNL4A,CYP2U1,DDX51,LOC100507316,MUC15,ETV5,EMC1,SRD5A3,ACOX1,DDIT4,CCDC11,CNPY2,RARS2,CBL,TXNL1,SREK1,LINC00665,SENP1,ALAD,TMEM56,SLC1A3,NOL7,TEAD2,JAK3,TMEM27,FYCO1,MYO5A,LINGO2,RP11-134G8.8,NR3C2,HSPD1,

	TMLHE,HECW2,GPR110,FAM26F,AFF4,SMR3B,ALOXE3,FANCF,RP1-93H18.6,KCNC3,TCAIM,MED8,BARD1,ETV5,ARHGAP27,LOC729970,GTPBP4,LOC101927550,PTPRJ,CCL5,SH3TC2,LINC00482,MYLK,EPHA1-AS1,CASC7,ELOVL6,ZPLD1,GPR126,HTRA1,ZMAT1,SLC10A4,EIF2B4,ELP2,MARS2,DCAF8,GSTO2,CHN1,BMPR1A,CPEB3,UXS1,ARHGAP19,GNAL,AJUBA,FTCDNL1,IKZF4,ZFHX3,HDAC9,RHOJ,AMMECR1,SSPN,TANK,PTGER4,SDCCAG8,PIP5KL1,PROSC,ZNF12,TEX9,LINC01186,DNASE1,WDR7,HSPBAP1,RP11-747H7.3,LOC642980,SAFB2,NLGN4X,DIAPH2,SENP6,CCDC93,SERPINA1,MSI2,ZNF789,CYTH1,CEACAM1,PDGFRA,PLTP,BRMS1L,CELF2,RAD51D,MACROD2,LIPA,LOC642852,NDEL1,KCNQ4,ZADH2,EXOG,LOC152225,HRH1,SOAT1,INHBA,VTI1A,PLCXD3,SPINT3,WNT6,SH3RF2,ARMCX5,ZNF750,SLC16A1,EMC3-AS1,C1orf53,PRDM2,PLEKHG1,KLHL5,ZNF302,PIK3AP1,ACER3,LOC101928152,SESN3,PHACTR2,RUNX3,VLDLR,ACER3,LOC286058,CLDN11,TMEM154,KIAA1715,IBA57,RP11-676J12.6,KATNAL1,KLF12,SREK1IP1,NTRK2,EPHB4,LOC100507634,KLHL15,TTC5,SLC25A17,LPIN2,CCDC85B,PPP4R4,RHOBTB3,MDN1,UNC5A,GUCY1B3,SPRTN,TXNDC16,FGFR1OP2,TRAF3IP3,SCN3B,PCTP,SYT1,SCNN1G,ZC3H10,SGMS2,ACER3,KIAA1715,MRPS7,APOBEC3F,RASAL1,ZGRF1,ZWILCH,PROSC,CAND1,ENPP4,DDIT4L,GM2A,GDAP1,RAB29,LINC00852,BAG5,FAM73A,SGMS2,TPGS2,PCK2,TIGD2,AURKA,DIAPH2,PCSK5,NRCAM,PLXDC2,RHOBTB3,MPHOSPH9,RP11-710C12.1,ST7-AS1,KCNAB1,ZNF610,CA3,DPP4,RP11-59H7.3,RASAL1,CALU,RP11-124L9.5,PDHA1,PRKCQ-AS1,COL22A1,ZNF594,AMOTL1,VTI1A,FAM122C,GAS7,EPHA1,CLCN5,NR4A3,COPS7B,PLCXD1,BCL2,KCTD13,HBEGF,STK17B,ST7-AS2,IDO1,RP11-1007O24.2,NT5E,MTMR3,ELK4,RANBP9,TMEM200C,TGFA,WDR36,ATP2A2,MLF1,LCORL,NMNAT3,RYK,DPP9,CCDC88C,FAM20B,SERPINB5,GPR87,GUCY1B3,CTC-471J1.2,SNAI1,TMEM97,ARL10,RMDN1,OR2L1P,ETS2,EHD4,ACBD3,KIAA1324L,OR2B2,FAM167A,RP11-554J4.1,MOB3C,ME1,UBLCP1,NAA50,TDRP,RPGR,ZNF182,SLIT2,GPX3,RALGPS2,HBEGF,TRAF4,MESP1,KLHL7,KANSL1,PTN,RBM34,PPARGC1B,ATP6V1C1,POLE3,FKTN,ZNF254,NPAS3,ARRDC4,KCTD13,AP1S1,CSRNP1,TRMT6,ME1,CCDC127,MOB3B,SLC46A1,NAV3,SRBD1,ELAC1,MTX3,SLC24A1,CBLN3,ZYG11A,PDE4DIP,GPR98,NANP,PINK1-AS,ZNF165,OSBPL3,CELSR3,GPR110,PBRM1,AC018816.3,LOC100131262,HCG11,IL1RN,DCAF7,PGAP3,FAM124A,MALSU1,CNIH1,SPTLC1,STS,CELF2,UBASH3B,SRSF1,NTRK2,LINC00881,IL1RN,DAND5,CNTRL,DDX55,XRCC4,METTL2B,NCDN,SPATA6L,GMEB1,TNXB,LRRC8E,IFRD1,RNF150,MCTP1,HCG11,SUN1,CELF2,KCNK2,IRAK3,RBM33,MPC2,MTMR6,FAM46C,ATP6V1D,AKR1E2,PDLIM4,GON4L,NBEA,TIMM21,CDADC1,ZDHHC4,PFKFB2,STYX,CYLD,PTEN,PAICS,CASK,BCL11A,PPARD,TGFBR2,SLC5A1,MTMR1,LEPREL1,AMBRA1,AHR,HERC5,RP6-99M1.2,AF086184,LOC283788,SPATA6,LOC101927841,SUFU,PICALM,TRAK1,SGOL1,BVES,SYNJ1,GPATCH2L,C1orf116,OCRL,CPNE8,WDR48,CMPK2,HSPA5,RIOK2,RAB

	11FIP3,FBXL17,HSPBP1,CFI,HSPA12A,RBM27,VEZF1,PTPRJ,TMED8,CTSS,EIF4EBP2,MTFR2,WDR11,PDZD8,DDX11-AS1,C12orf66,EIF2AK3,GFPT1,CCDC181,RP11-687F6.1,EFEMP1,LEPROTL1,TNIK,CELSR3,SOCS6,EIF2B4,STS,HRASLS,SNW1,CAMSAP1,PLXDC2,RBPJ,ATP6V1C2,MAGI2,COL4A3BP,KCNJ16,ITGB1,RNF150,VRK1,AP4E1,CDC14B,KLHL29,KCNMB1,EIF4E,TSHZ2,MCTP2,ARHGAP29,NADK2,EMR2,RORA,MBD2,CAMK1D,SFXN2,RHBDD1,PIK3C2B,STK38L,CCSAP,RAPGEF2,RORA,ALOXE3,SEL1L3,TDRD9,TVP23C,STS,WDR61,AURKA,NEK5,LCLAT1,KIAA0355,CTA-445C9.15,GPR126,ZNF347,ACVR2B,SLC44A3,MDM2,THUMPD3,FUT8,HMMR,ERGIC2,ABCD3,SP4,ZNF275,CAMSAP1,PI4K2A,USP2,PLA2G12A,TOR1AIP1,RALGPS2,COPS8,GSTA1,GYG1,KIAA1549L,RP11-443B7.1,PIGW,MAPK9,PRSS23,PHYH,TMEM92,EFCAB14,ZFP28,EAF1,CCL5,SLC39A9,CMYA5,SP100,PSEN1,ZNF677,RFC3,ZNF337,CDC14B,RFWD3,SUSD3,BTBD9,NPAS3,ERVMER34-1,GAN,LOC101926915,TFIP11,IRAK3,TSC22D2,CCDC125,PHF20,EGLN3,USP28,FANCI,ZNF789,CDC27,IPO5,MUC20,NAAA,KDM5B,LIPG,PPM1D,PYDC1,CAMK2N2,HM13,RP1-30M3.5,GPR115,P2RX5,IL1RN,DDX18,RASGRF2,OXR1,HIGD1A,TNFRSF11B,PIR,RP11-214N9.1,DSE,MPP5,LOC102725383,PINLYP,LOC101928647,BCL6B,SLC31A1,TBC1D12,SH3RF2,FKBP14,CABP7,ZNF275,IKZF2,RP3-388M5.9,HABP4,PIGM,ITGB1BP2,CDCA2,HADH,GTPBP8,MGC2889,XYLB,PELO,SETBP1,TTC21A,SPRY1,NEU3,FBXL4,EGR3,NTN1,NDUFS1,MTDH,DYRK3,PROL1,ZBTB20,ZSCAN12,CDC42EP3
E-BCSC_DOWN (FC <1/4 in CD44-ALDH+ vs CD44-ALDH-, GSE52262)	PPP1R3F,STAMBP,TSGA10,ERCC6,FLT3LG,C1orf21,C5orf66,ADAM9,C17orf97,PREX1,MIR5188,PLEKHH1,TLN1,MEX3A,L3MBTL1,C1orf216,GTSE1,SNTB2,HIST1H2BN,DQ592442,COL27A1,NLRX1,PDE4D,NAA30,EGR4,DMXL2,_MARCH1,SOD3,TSTD2,FAM198A,PRSS54,IQCE,MCF2L,HDAC7,ZNF107,EPOR,BIN3,UCP2,MTO1,ALOX12,NUSAP1,TTC22,MRPS5,FRMD8,CHST2,CYP4X1,ARHGAP29,SOX12,COL9A2,SNX24,TACSTD2,MSX1,CHST11,LOC102724967,DQ576994,LINC00968,FLJ31104,CLEC12A,TNFRSF10C,IFT43,RP11-464F9.20,DNAJC7,PCBP2,BMP4,FOXN3,RP11-650K20.3,GAA,RP1-151F17.2,MORF4L2-AS1,RP11-214K3.19,COQ10B,CD101,KMO,RP13-638C3.2,GRAMD3,ZDBF2,NUMA1,MICAL2,LOC102724975,SAMD5,MTFR1,MUC4,CIRBP,RRM2,ANPEP,SIAH1,TFDP2,TROAP,IFNGR1,NCF4,CYP4B1,FGG,RBM8A,NANOS1,CDC14A,SYTL2,CAPN13,PABPN1,CARF,SSH2,WDR52,TCP11L2,ZNF141,TMCO5B,RHOJ,RAB3GAP2,WWC1,CCBL1,TRNAU1AP,RBM5,SCGB3A1,NPL,PGR,PON2,FLJ13773,TMEM176B,SIX3-AS1,_MARCH6,KCNE4,C5orf42,HIST1H1T,RECQL5,GDF15,PDLIM5,KCNJ13,WFDC2,MLLT6,SETMAR,LOC102725022,LIN7A,RXRB,C9orf47,CARS2,ERBB3,SNORA74A,PPIL2,KMO,AP5M1,C17orf104,B3GALT6,DPH6,AX746968,_MARCH8,RNF213,AMIGO2,MSL3,CDK6,SDCBP2-AS1,LINC00877,KLHL29,NCOA2,UBE2G2,CECR7,TAS2R14,FAM104B,WHSC1,HNRNPA1,AC002059.10,BDH1,AF131215.8,LOC645513,C4orf19,ANKDD1A,VPS8,PRKCD,FAM196A,ITGB2,CEP57L1,FLJ38576,I

	TGBL1,RNF43,AKR1C1,SNORA74A,KLF9,PIGX,RARA-AS1,SNX32,GM2A,ZER1,ZNF518A,CLEC2D,GPX2,YWHAH,SEC31B,SCN8A,AKAP12,ZNF81,U91328.2,INPP4B,NAMPT,SLC37A4,RASSF9,ASIC4,LOC101927377,SLC44A4,PLEKHA6,DGCR11,GINS2,TRA2A,RP11-559M23.1,ID4,RAB18,ZBTB44,CP,ITGB8,SPACA6P,TBC1D22B,ZNF239,LOC100128079,DIMT1,LA16c-395F10.1,EFHC1,BUB1,HIST1H2AM,LOC101926944,TRAPPC10,PCDHGB7,SRGAP1,CEP78,DCHS2,TMEM86A,MLPH,SYTL2,LOC100287290,TMF1,FAM149A,RP5-894D12.4,PMP22,SQSTM1,STARD13-AS,S1PR3,PDS5B,MIR205,PCNXL2,DTWD1,CMBL,C11orf72,AKNAD1,TBX3,MAST4,DQ594366,MPDZ,LRP2,NHSL1,TANC2,OSER1-AS1,LINC01220,B3GALT5,LOC101928560,RP11-474D1.2,ERICH3,PPP2R3C,RP13-122B23.8,VPS13C,DOCK4,ZCWPW2,BRWD1,STEAP4,IRS1,LOC100996756,PIK3R1,ZBTB10,CLSPN,HEXIM1,PPP1R3C,MFSD4,DLX4,TP63,PEG3,ZMYM3,DGKE,ACADSB,CFDP1,WTAP,INSM2,WDR5,TM4SF18,RP11-480I12.10,FAHD2A,MAOA,LOC541472,SORBS2,ODF3B,CREM,WDR74,SDC3,DSTYK,BLZF1,LBP,AC079741.2,ITIH2,LINC00441,AXL,RBM26,LOC654342,RFTN2,IGFBP5,ECM2,SCARA3,NLRC5,SNORA74A,ARHGEF12,RP1-68D18.4,RP3-368A4.6,ARHGEF39,RP4-798A10.7,MUC4,CFB,RPE,LRRN4CL,AKAP12,SFMBT1,BRWD1,TBX3,PATE2,MS4A7,NHP2L1,UTS2B,SYCE2,ZNF589,TTC22,RP11-355F16.1,TBX3,NRG1,WDR38,SF1,CLEC2D,POM121L9P,CTD-2287O16.5,ENO1,WDR31,NFYC-AS1,HIST1H2AK,BBX,SLC25A4,ABCB1,RUFY3,CYP39A1,ASXL1,RP11-199F11.2,NXPE3,ZNRF2P1,PIK3R4,PTPN2,CAPN8,DNAH5,RALY-AS1,CEP19,APBB2,CDKN3,FLJ38773,NRD1,AKAP10,LBP,AP4B1,VAMP1,CD99,EDRF1,EIF2B5,KIAA1324,C2CD4A,CTTN,CLU,LOC100505564,ABAT,AC016999.2,ADPRM,RSBN1,C18orf42,TBXA2R,GATA3-AS1,APOF,GALNT6,MAF,ADAMTS9,ARRB1,SMIM14,MOCS1,OAS1,SCAI,NPR2,LINC00664,CD44,CSF3R,SLC7A2,LINC00115,PLA2G12A,SEMA6A,PPP2R1B,DIO2,FBXO9,AK021933,AGR2,NDUFV2-AS1,HIST1H3C,REEP1,HOTAIRM1,SFXN2,TPTEP1,ALCAM,KDM4C,TMEM223,RASSF10,DNAH12,BCL2L14,HSPA1L,FOXP1,RP11-112J3.16,GRAPL,PHKG2,GALE,LOC101928614,WDR96,ID1,ABHD5,LACTB,SOX8,DIO2,MYBPC1,PNISR,SCAND2P,DLG5,RP11-1275H24.2,FLJ46875,LOC100506235,MUCL1,FGB,AR,CDC7,ESCO2,CLEC7A,PCSK7,TSEN54,C6orf141,GXYLT2,ZNF483,STK16,SPARCL1,THRAP3,SNTB2,MUC4,GLUL,AR,DNASE1L1,CHSY3,RGMB,EGFR,AXL,SLC16A1-AS1,LOC101927038,BC045784,SEMA3C,NAA25,DNAJC6,ZNF784,GPR22,FAM47A,YWHAH,KRT222,LCMT2,POLE2,LOC730101,GANC,COPS7B,TFAP2B,FAM69A,DIRC2,KCNJ8,PSTK,EDNRB,TRDMT1,SDR16C5,RP11-350F4.2,FGFR1OP,EFCAB6,PEX6,LOC729870,HPX,GALE,LPCAT4,FRYL,TGFBI,SEMA3C,PTPN2,OFCC1,CD44,KIAA1755,ALDH2,RP11-210M15.2,KLF8,RP11-1137G4.3,DBN1,SLC40A1,LOC101927027,C8orf4,SLC27A3,MFSD4,VWC2,SNCA,KIAA0101,XRCC6BP1,CPSF6,CDO1,CLK4,RNF152,CHD2,DLG1,FRAS1,FAM120AOS,SYTL2,LOC101930067,HOXC10,OGFR,TMEM143,TBRG1,AFF3,PDE7B,PDCD

	5,RP11-196G18.23,AX748273,AK055458,FBXL12,KIAA1324,RGCC,GALM,FAM150B,ZNF214,RP11-506N2.1,OSTM1,AREG,EGFR,ROR1,SPP1,ZNF175,IL1R2,ANKRD30A,STK17B,CTA-390C10.10,FAM171B,ADAM30,MUC6,AK9,FAXC,GJC1,ALDOB,MS4A7,RNF213,BRWD1,KATNBL1,NDUFB1,FILIP1L,PCDH9,RPS24,GJC1,FREM3,CARS2,TSC22D1,KRTDAP,SYT7,ZC3H4,TMEM134,SYNJ2,CLEC1A,OAS1,TFF1,HIST1H4A,SYNPO2L,MESP1,LOC692247,WDR59,ZC3H14,MLLT10,FUCA1,ZNF571,TTC17,MGC12916,LOC100128108,AK021933,STK4,CTD-2033C11.1,IFFO1,LOC729658,ZNF440,WTAP,C4orf27,DGKH,RP11-63A11.1,HSPD1,AMT,TFAP2B,CHPT1,AREG,ATP5C1,LEF1-AS1,PLAC8L1,PTPN13,TFAP2B,IL1R2,TMX4,TOR4A,CNOT6L,CCND3,CHD2,HS3ST4,FAM24A,FAM20A,LOC102724809,LINC00280,MS4A7,TSC22D1-AS1,TMC5,RP11-466P24.7,TNPO1,FLJ16734,AGR3,RP11-138I18.2,RP11-138I17.1,PCDHGB8P,GRK6,TMEM176A,TSR1,FTCDNL1,FAM3B,MCTP2,FMO2,CLIC5,PPME1,IGSF5,FANCC,HIST1H3B,STAT1,LINC01138,EME2,TCEB1,TFPI,FMO2,ZBTB46,MST1L,C18orf61,EPHA10,DENND4B,TFPI,CTD-2302E22.4,TUBE1,IPP,CLOCK,AC092660.1,FAM193B,ODF3B,DAPK1,FADS2,SALL3,MCM3AP,CCDC158,VSIG1,LOC101926967,CHRM1,PLA2G4C,CD93,CROT,COL22A1,BNC2,SLC23A3,SPP1,TFPI,EPOR,LOC101929511,NMRK1,FBXO7,ZNF283,WDR63,MECOM,GNA14,FGFR1OP2,E2F1,BTBD11,GATA4,KIAA1430,AGT
M-BCSC_UP (FC >4 in CD44+ALDH- vs CD44-ALDH-, GSE52262)	IGJ,MMP3,IL2RG,IGLC1,IGHD,ADTRP,GPR110,SDHD,LINC00158,PTHLH,SLAMF7,XXbac-B476C20.9,EML6,IGLV1-44,MMP10,NFRKB,IGKC,LAMB1,IL13RA2,LOC100132319,NTN5,UHRF1BP1L,CLMP,FGD6,GRP,ZNF502,SEMA6D,FAM101B,RAB39B,PDGFRA,ZFR2,MIR100HG,CCDC40,AOX1,GPNMB,LOC101929109,LINC00294,LINC00957,FAM73A,MEDAG,ACTG2,CASD1,RHNO1,RRAD,PLD1,BASP1,WDR66,LOC102723479,JAM3,COL3A1,A2M,AP001462.6,STK24,PET117,COL6A3,PHKA1,HTR2A,ZBED5-AS1,DCAF13,KCNMB1,PAMR1,COG6,DKK3,MMP1,QSOX2,FGA,ZNF672,NID1,MMP12,CWF19L2,NRCAM,KCNE4,RP11-757F18.5,APOD,FAM13A-AS1,DNAH5,FHL1,PANX1,ZNF570,NAP1L2,SRGAP1,CALU,IGHM,KCNK1,SCG5,HTRA4,PPIL1,TIMP3,COL14A1,SMIM7,TATDN3,ARHGAP24,FEZ1,MME,C2orf44,DCN,PDLIM4,MMP2,FGF1,CDC42EP3,LCA5,GSTO2,IGLL5,TWIST1,MXRA5,GEM,ARNT,MCM5,RHOJ,RASIP1,CEP89,COL17A1,PRRX1,VPS26B,CHST7,NCF4,SPRY1,ZNF426,NEK5,IL1B,SLC16A1,CPB1,TP63,ISY1,CDCA3,FGG,TNS4,ADAMTS1,DPYSL4,NEFM,AXL,AC007362.3,HTRA1,TRMT6,ZNF275,CTSC,KBTBD2,OCRL,FGB,CXCL13,TTC31,PSMB1,SMYD2,RIMKLB,COL4A1,DIRAS1,SMC1A,IFI35,SP110,CIAPIN1,ZNF775,SERPINB5,MID1,CEACAM6,PPP1R13B,POSTN,STX4,PARP12,RC3H2,AMOTL1,CCNJL,SUFU,ARMC7,CEACAM5,NPTX2,BMP2K,SERPINF1,GJA1,C16orf95,PRDM10,KIF3C,MEG3,TFIP11,MTSS1,SPINK1,ADAMTS2,TMTC1,GZMB,AGPAT4,CCNA1,CCDC11,ITM2A,PLSCR2,INSR,TGFB1I1,LRCH3,PSMB7,HAS3,NT5E,AMIGO2,PROS1,HSPA4,OXR1,ARL10,PDPN,SMKR1,TRERF1,HHAT,GPR125,LOC102723845,GCFC2,LBH,

	CERS6,FJX1,EFCAB2,PROCR,ATG5,COG7,ENO2,HCN3,SLC44A3,GPC1,VAPB,THAP10,VWA5A,KATNAL1,ERG,ATG16L1,MFSD9,SYTL5,DIEXF,CDS2,IREB2,SLC29A2,ZNF697,PTGER4,THBS2,RBMS3,TMX4,AXIN2,ACTA2,NETO2,KIRREL,C17orf100,SCML1,STON2,SNN,SLITRK6,GPC3,NAA30,PARD3-AS1,KIAA1715,C14orf132,DIP2A,TMPO,LOC101928554,EFEMP1,SETD7,HGF,GALNT6,RASSF6,LAMA3,TUBB6,STC1,SAMD5,EIF5A2,ARHGAP23,EIF2S1,KLHL6,ZNF185,LRPAP1,TMEM234,ZCCHC7,CMYA5,NRP2,AHNAK2,CPE,RP5-1136G13.2,INHBA,SPAG9,CASP14,RAB27B,TDRP,COL5A2,CTD-2124B8.2,CCDC127,FST,SKIL,THEM4,BMPR1A,ZNF616,LAMA1,GAS2L1,IFT52,GCC2,CELF2,RHOBTB1,FHOD3,TRAF3IP3,SLIT2,PTGS2,ACPP,IRX1,ZUFSP,TRAK1,PDS5A,IGHG1,BVES,SAYSD1,ARHGAP36,KLF12,RRN3P3,BEX5,ADAMTSL3,CRY2,CPT1A,LOC100131262,SAMHD1,ITIH2,TLCD2,FBXW5,LOC439911,SRPX,UBE2N,PTAFR,GPR176,SRRD,IL18R1,RGS1,RGS5,SP4,IL24,CHST11,MTX3,SLC39A11,SUN1,PAPPA,NRG1,C9orf156,ARHGAP25,TMEM182,ZNF641,EDNRB,EPS15L1,SMARCA1,TFF3,CD200,CUL1,HMMR,PLAT,NOTCH4,SCGB2A1,SCRIB,PEG3,IL1RL1,SSPN,HP,COL1A2,COL12A1,DRAXIN,SPARC,CCND2,DSTYK,JAG1,FMNL3,ZNF222,PIGN,C2orf40,TMEM127,OXSM,H2BFXP,KIF5C,SMOX,C5orf63,GRSF1,STS,KIAA0040,PENK,LCP1,HCG18,ABHD6,TMEM167A,ZNF214,NID2,LYPD6B,DYNLRB2,CPNE8,IGFBP2,PRKD1,RRP15,USP47,ETS2,TAGLN,KBTBD8,BICC1,CYP2U1,SULF1,ACSS3,GGPS1,BCL9,MICAL2,TFPI2,SRD5A1,PKLR,SH2D5,KIF3A,LINGO2,EIF2AK3,COL4A6,GCNT4,SNAI2,RRAGC,WDR36,HERPUD2,SBSPON,ZDHHC16,HM13,SERPINB9,SLC7A7,C8orf88,CDC37L1,HCLS1,POLD3,LHFP,GGNBP2,OXTR,BNIP3,OPA1,MLF1,CSNK1D,PEX1,SRGN,COL4A5,RRP7A,DPP4,TMEM154,AFF3,PELO,CD109,GOSR2,EHD4,RPUSD3,ERVMER34-1,PI15,TSHZ3,ZNF165,TRAF1,SLCO3A1,MIR31HG,NTRK2,FUT1,SLC25A4,TFPI,DGCR2,SIRT5,PLXDC2,USP2,PTGS1,WDR89,APOE,CINP,SOCS5,LOC338667,RHOBTB3,IGFBP1,ZCCHC14,ZNF7,SPATA6,SRGAP2,MYLK,STK38L,UNC119B,CLIC6,SEL1L3,AK021804,SLC16A7,FKBP3,ANKRD22,TMEM178A,SOBP,CTA-445C9.15,KLHDC8B,SLC24A1,SH3RF2,KLHL15,PIK3C2B,HDAC9,SEC63,C3orf17,WDR47,JAM2,TFCP2,WDR7,TMEM144,CLCN6,NDUFAF4,FYN,BMP5,SAMD12,ZHX1-C8orf76,CNOT6,DCLK1,STEAP2,VTI1A,FNIP2,ITGB3,HIGD1A,PCNX,UEVLD,LRBA,TMEM170A,LOC100288911,CDCA4,IRX4,NAT1,FAM171B,POP1,METTL2B,RP3-428L16.2,PIGW,GPD1L,ISG20L2,EPM2A,PTPDC1,ATP11C,FAM167A,TXNDC2,REC8,UCHL1,KIF21A,LY9,C1orf53,FAM126A,ITGBL1,ZNF101,RHOH,FN1,GM2A,FAM175B,BEX2,ADAMTS5,MRPL2,C7orf63,PLCB1,LPAR1,RNASE7,ARMC1,MCM3,AMPD3,CAV1,FMOD,OSGIN2,PHF20,TUB,ACBD3,RASSF8,LOC101928487,A2M-AS1,TGFBR3,HMGB3,COL8A2,C1orf112,COL2A1,GNPAT,HRH1,RAI2,LINC00888,AF086184,UQCRB,TBC1D22B,LIPA,TMOD2,NAV3,AP1S2,HMGA2,STK39,PLEK2,SERPINB9P1,SERPINB8,CDH11,ARID3B,DIO2,MTMR6,CASC4,GAL,PPP3CA,TAF3,IL20,STX2,CATSPER3,PAGR1,HIVEP2,ITGA6,TAC

	1,RSPO3,SEC22C,COL4A2,ZGRF1,PFKFB4,NTRK3,HBEGF,SLC7A2,ATP8B2,ACTR3B,ZNF75A,MRPS30,PGAP3,SERPINA1,ALOX15B,YY1,DHTKD1,PTPRU,MSRA,DGKA,EPS8L1,ZWINT,ZADH2,PRICKLE2,BHLHE41,SLC35B4,CDS1,STXBP5,RPL18,CITED1,ZBTB40,PLEKHG2,TMEM237,WLS,PPME1,FAM46C,RASSF3,UMPS,IPMK,APBB2,SEPSECS,TGFA,TSN,ADAM19,MIIP,SETDB2,LOC284023,CD99L2,PLCD3,ECI2,PRUNE2,PRMT9,EPHA1,EGLN3,NRP1,AGTR1,SLC26A10,DNAL4,AURKA,RASAL1,LY6K,ZNF302,ENPP1,ANGPTL2,TCEAL2,RPP30,MRPL35,AOC2,CYTIP,KCNMA1,SSX2IP,NSD1,ALDH4A1,EHD3,IMPAD1,BCAS4,FBXO28,SYT9,CAPRIN2,SCGB1D2,SLC30A1,BCAT2,ETV5,ZWILCH,RIOK2,PARD6B,DNAJA4,PCSK5,PLAU,KLHL42,MMAA,LCLAT1,_MARCH9,TRAK2,IQGAP2,FAF1,SCUBE2,ANAPC7,INF2,MSRB3,FBXO40,FAM20B,MYLK-AS1,SPATA2,DFFA,LRRC8E,LINC01133,HS3ST3B1,TDRD9,CSRP2,TP53TG1,FAM174B,BRD2,C15orf41,TIMMDC1,FSIP1,IRAK3,ACACB,WIPF1,LOXL2,DNALI1,SDC2,SHROOM2,RP11-59H7.3,EMB,GPX3,FGF2,MARVELD3,HTRA2,PTPRJ,CHMP4C,GPATCH2L,AQP9,TRAF3IP1,SNAI1,PPP1R2,C12orf4,ZNF512,ATMIN,ITGAM,ZNF419,LPCAT2,B3GNT5,C1orf74,POU6F1,SLC35D2,IL17RD,RGS12,LOC100499489,STEAP4,DST,PTEN,DCLRE1B,PLA2G16,PRKAB2,LAMA2,TCEAL7,PRSS23,NUDT5,ITGB5,TBC1D30,G3BP1,TOMM40L,SLC38A6,PRKAR2A,JAK3,GNB4,TENM2,CPT2,C5orf51,NDUFAF7,MLKL,FAM134C,GPR126,USP46,ZNF836,FAM208B,TNIK,NEGR1,PLXNC1,TCTN3,SLC7A8,RASL11B,CEP63,WRAP73,CTSL,METTL1,STMN3,PSME3,MYO10,BCL2L2,SYNC,KIRREL,GGT5,HAS1,EBF2,SPATA7,CPQ,FAM26E,ANKRD50,S100A13,PDLIM5,ADAMTS15,RNF144A,PDLIM4,FAM65C,PLD1,LINC00478,SYTL4,AXL,GNS,FBXO17,ACO1,ACOX1,SHC2,HLF,FZD10,VAPB,GSN,UNC13B,CORIN,HEBP1,ADAMTS8,SRGAP1,LRRC49,PTGDS,NIPAL2,CKAP4,KANK2,KLHDC8B,AGAP1,MRC2,SLC22A17,JAM3,LRP4,PER3,TMEM5,PRDM6,MARVELD1,HOXC9,RGN,NR1H3,TK2,TMTC3,F10,LOC101927263,RASSF9,TSPYL5,GUCY1A3,ZBTB47,SELM,MYO1D,CAMSAP2,RP13-39P12.3,C8orf88,DHRS3,TSPAN11,FAM8A1,KIAA1324L,HSPA12A,DOK5,CD59,KDSR,WDFY3,HSBP1,CRYAB,ZDHHC15,PFKM,GUCY1B3,LMLN,GYG2,GLI3,ADSSL1,SNHG18,MAP1A,CAMK2N1,RBP1,GALNT5,ADCY9,CC2D2A,DPCD,MGC24103,PLTP,TBC1D16,LEPREL2,FAXDC2,WDFY3-AS2,TMEM117,SORBS1,SBF2,FAM198A,CST3,TNFRSF19,SLC44A3,GAS7,RNF150,FAM13A,CTSF,IFT22,LPP,NR2F1-AS1,HDAC11,NHS,HSD3B7,FAM229B,UACA,SLC39A14,KIF13A,RP4-758J24.5,CLN5,SRI,ANKRD29,SCARB2,WASL,CAPN5,CNIH3,EDNRA,C11orf95,LOC100132891,CARD10,THSD4,SLC25A43,TRIQK,TMTC2,KIF26B,GSTM3,PSMD12,ARL6,PRKG1,LDOC1,LRRC32,ABCB5,RRBP1,BBS9,SLC38A6,ALDH7A1,MSRB2,HOTS,KIAA1841,FAM127A,LPHN1,CTBP2,ARL1,CCDC127,TMEM158,PLA2G4C,FUCA2,PLBD2,ARPIN,NBEA,NPAS3,DIAPH2,RP11-134G8.8,DDIT4L,HSPA12B,LYVE1,ZNF826P,KANSL1L,FGF2,REPS2,PAQR7,TLR3,FBLIM1,DHRS7B,MAN1C1,TMEM55A,SPIRE1,KHDRBS3,CX3CL1,LOC100507165,UBQLN

	2,CORO2B,BDKRB2,XPNPEP2,GNAQ,LOC100287387,PTPN14,NRP1,LRP3,TAGLN,MPZL1,KIAA1377,SNX16,MBTPS2,LOC729970,HIGD1A,RIMKLB,AK5,COPS8,LLPH,ZDHHC9,UBE2E2,EXT2,SPTSSA,TPSAB1,SPESP1,GNG11,KDELC2,SMARCE1,TMEM220,FAM110B,YAE1D1,LOC101927943,NUAK1,SPATA6,TSPY26P,ZNF697,NLGN4X,CRTAP,C1orf56,IL6ST,SLC16A14,DOK6,SDC3,CERCAM,ANPEP,ETV1,ADH5,IL20RA,ABHD12,PRDM5,EVC,LDB2,MBD5,TGM2,MDK,RILPL1,APLP2,PLXDC1,C3orf80,OAF,PI16,RGMA,COPRS,TBX15,PLOD1,DPY19L4,C1orf198,AIG1,ATP6V0E1,LGMN,SETBP1,C7orf63,NRK,TMTC1,EXTL2,TIMP4,HCG11,ROR2,HIBADH,PGRMC2,ITGA11,SLC25A3,TMEM17,LEPREL4,TCN2,MIPEPP3,METTL21B,PYGL,CPM,SLC28A3,LOC100506119,CREB3L1,ENPP1,CYB561,B3GNT1,CYP27A1,LGR5,SLC1A3,HDLBP,IPP,ACE,AGT,FLNC,KCNJ2,STON1,ACSL3,IQCK,DRAM1,ARMCX4,C1orf216,TMEM165,FAM46A,ETV5,FAR2,ZFHX4-AS1,NFYB,NACC2,NPEPPS,ALDH6A1,C1orf21,PHLDB2,GPC1,IL13RA1,TCF7L1,MBNL2,HUNK,NEK11,NPR2,EEA1,MAGED1,FKBP1B,NKIRAS1,FLCN,SLITRK4,SLC37A3,NRXN2,TNFRSF12A,C9orf3,NENF,CCL11,LOC646762,FBXO28,LINC00922,PCDH19,KLHL13,HOXD-AS2,CNTLN,RP1-78O14.1,MKX,SNX13,NALCN,C6orf120,CDK2AP1,IFT43,NEO1,TGFB3,ZNF300P1,DCUN1D3,LMCD1,PC,RAB40B,_SEPT8,CLCC1,DHFRL1,BMPER,STXBP5,QSER1,PXYLP1,UBTD2,TMED4,MMP19,THAP10,EBF3,CTB-12A17.3,SORCS2,PRRX2,MTFR1L,VAT1,DAPL1,EXPH5,SOCS2,SUN1,FAM162A,TANC2,MNS1,LYPLAL1,ABCA1,RP11-403P17.3,CHST3,EHD2,MYO1E,ASTN2,SAV1,OGFOD3,LTBP3,ARMCX5,C11orf24,ZBED3,SH3RF3,RUNX2,INSR,IGFBP7,CAND1,IFT80,C8orf48,CCDC8,BMP4,MORN2,PRPH2,TMEM54,BLVRA,C11orf49,RADIL,PGRMC1,MAGT1,SWI5,PRDX4,SCARF2,CTIF,CNIH1,TNKS1BP1,CD34,VKORC1L1,PCDHB15
M-BCSC_DOWN (FC <1/4 in CD44+ALDH- vs CD44-ALDH-, GSE52262)	EQTN,ADAM9,SRRM2,TTC17,RBBP4,LOC729870,CXADR,VCPIP1,ARHGAP28,ABHD11,SNORA71B,MFSD8,ZNF33A,ERVFRD-1,CRYZL1,CDK12,WBSCR27,SMOC2,PDK3,LOC101930657,DHX30,STXBP2,PLA2G12A,ZNF397,WSB1,KMO,MGAT3,SLC11A2,LARP7,CDK1,LOC101927018,LINC00441,ZNF229,KCTD4,MCF2L,MTHFSD,DQ594366,C17orf104,RP11-464F9.20,NFYC-AS1,LMF1,MAP3K13,C1S,DNM3,LOC100507616,CHORDC1,HLA-DRA,ZNF518A,DAB2IP,SIX2,RNF141,HIST1H1T,HIST1H3G,RP5-894A10.6,SLC25A27,LOC102724387,AGAP4,OCR1,DIAPH2-AS1,REEP1,CHI3L1,DTD1,FAM3B,SYNJ2,C5orf28,RFX3,LOC101928483,PCDHGB7,INTS10,KDM5C,OFD1,MTHFD2L,FAM149A,RP13-122B23.8,ZFYVE16,DAPK1,ASIC4,HSPA9,VGLL1,KIAA1919,PWAR6,DLEU2,MLLT10,IFNGR1,WDR26,RBM47,CDT1,AMBRA1,ARHGEF12,CNBD2,GOLGA3,OR7E47P,HIPK2,SLC26A6,RP11-158G18.1,THBS3,IQGAP3,USP22,PPA2,PDK1,CTD-2196E14.6,SH3BP2,RUFY3,EGR4,PDS5B,LBP,FOXN3,TMEM143,C11orf31,UBAC2-AS1,TAF15,SNHG19,CTNNB1,ARHGAP29,PCDH9,ERBB3,ELMOD2,PCBD2,RPS24,INTS6,GAS5-AS1,PPM1A,FLJ31104,YWHAH,ALDH8A1,RGMB,RNF213,RP11-676J12.4,LDLRAD4,HECTD4,GATA4,RORA,CD44,AF19844

	4,LOC100507217,SRSF12,RHOF,PRICKLE1,SLC10A1,ATP5S,ZNF117,TMF1,CTA-292E10.6,TTC28,SETD9,DLX2,FLT3LG,CA1,PRMT2,SORBS2,SYCP3,ICMT,IL1R2,GPD1,SYT7,HIVEP3,FAM20A,ZBTB10,PPP1R3C,PCDHGB8P,MINA,SATB1,GALE,HIST1H3A,LOC101926967,MUC4,AC092192.1,KCNJ13,CYP39A1,PRSS12,FNBP1,CDK13,AMMECR1,RP11-196G18.23,CFLAR,CD47,TTC22,DNAJC7,SLC25A37,C4orf27,XRN1,DCAF11,SEMA6A,LIPE-AS1,HOXA6,FRYL,EIF4G2,RP11-559M23.1,EDRF1,NT5DC2,PDCD4,ZER1,KIAA0922,SCN8A,SNRNP200,LOC93432,SDCBP2-AS1,RP11-334C17.5,LOC158960,SCARA3,ARHGEF39,TSC22D1,QSER1,RP4-798A10.7,_MARCH1,ZNF571,SNORD8,LOC100506379,DNAJC6,ODAM,CHPT1,CENPU,ELK1,UBA1,RP11-138I18.2,KLHL10,CIRBP-AS1,SLC6A16,MUC5B,MARK1,ID1,DTNB,WDR59,SUSD4,STK16,WFDC2,FLJ46875,SEZ6L2,RP1-151F17.2,FAM150B,RP13-638C3.2,LOC100129112,FBXO9,PTPRO,PDCD5,TLN1,NR2E1,MAP3K15,BCL2L14,IL12RB2,COQ10B,RBM5,GSK3B,ZC3H4,ST8SIA1,LRRN4CL,TECR,ODF3B,RP11-480I12.10,PON2,CLU,AREG,GLUL,PRSS58,U91328.2,PSORS1C3,SOD3,CHSY3,GUSBP1,SMIM14,DCHS2,LOC102723886,PIK3R4,CTA-390C10.10,MIR3916,KRBA2,C3orf65,MGP,GRAPL,TNPO2,PROL1,LINC00472,CTD-2302E22.4,RALY-AS1,FOXP1,TRIOBP,COPS7B,CTBP1-AS,ZHX2,B3GALT2,INSM2,PRPSAP1,LRP2,DGKH,RP3-368A4.6,ANKLE1,LOC100287290,MBTPS2,ANGEL2,IPP,PPP1R1B,PADI2,WDR5,TOR4A,C11orf72,ROR1,CACNB2,SOX8,SRSF3,BC045784,ZNF440,FBXL16,CNOT6L,ROPN1B,ALCAM,TSGA10,HIST1H3I,RAB26,KLF8,TFDP2,GLIPR2,FUS,FTX,BBX,AF520793,PITRM1,HIST1H2AM,LOC101928968,LINC01138,FILIP1L,GUCY1A3,NAPSB,ADAMTS9,CTD-2033C11.1,CLEC7A,RP11-499E18.1,NCSTN,AREL1,OFCC1,MYCL,EP400NL,SNX8,KIAA0907,AL832163,NRD1,RP11-355B11.2,NKTR,EME2,DOCK4,MST1L,SLC27A3,OR7E156P,LOC100505564,AK055458,ELF5,XRCC6BP1,RP11-85A1.3,ECM2,FGFR1OP,LOC101928560,DQ592442,SCAI,LOC101928140,KIDINS220,ZBTB44,ABHD5,CNKSR3,FABP7,TUBE1,TRA2A,HIST1H3C,ROPN1,PDZD2,LPCAT4,CLEC1A,CIITA,TSTD2,MFSD4,MGC12916,FANCC,WTAP,LOC101928614,AK021933,PLA2G4C,HIST1H3B,FMO2,EGFR,ITGB8,CCND3,KIAA0101,C4orf19,RGCC,CXCL2,PDCD4-AS1,SNORA68,ZNF283,KIAA1755,TSC22D1-AS1,RPS27,FBXL12,MCM3AP,HS3ST4,TSR1,CLOCK,CARS2,HIST1H4A,NMRK1,FTCDNL1,SLC23A3,CHRM1,EPOR,SNORA74A,BTBD11,KIAA1430,LOC101929511,KANSL1,JADE2,GPR174,PTPRC,DLG1,RCAN3,CARD11,TTC7A,SCARNA17,SATB1,LOC101928173,DENND3,NR3C1,DENND2D,AF007147,SYNE2,JMY,_SEPT1,PTPN4,ICAM2,FASLG,FAM159A,POU2F2,DOCK10,INPP5D,SH3TC1,ADAM8,SLAMF7,PDE3B,PRKCB,RASGEF1B,NEDD9,PRF1,SPOCK2,LOH12CR1,GPR132,PLCG2,MGAT4A,KLF12,DDX17,CD160,LOC727820,IL4R,LOC145474,MZB1,KIAA1551,STK4,CXCR6,ATP2A3,CYLD,EAF2,KCNA3,SCML4,SLAMF6,SYNGR3,GIT2,PTPN22,LY9,PCNX,TUBA4A,STK17B,ITPKB,SEL1L3,SLAMF1,PRKCQ-AS1,PTGDR,GUSBP11,CD79B,STK17A,DOCK8,M

	GEA5,CD38,IGLL5,MCOLN2,ARHGAP25,TRAF1,SUSD3,CDC42SE2,CD226,SLC38A1,FERMT3,SMAP2,RGCC,ITGA4,SP100,RP11-489E7.4,IRF4,LAG3,GLCCI1,TAGAP,KIF21B,PIK3R5,DENND1C,KIAA0922,ITGB7,HLA-F,PDE4B,FAM65B,PIK3CD,CNOT6L,PASK,GFI1,CXCR4,XCL1,SEMA4D,IL16,AIM1,RASAL3,GBP5,VPS13C,DUSP2,C11orf21,TRAC,SYTL1,IKZF2,WIPF1,TRAF3IP3,_SEPT6,ISG20,PRKX,CD79A,MYO1G,CLECL1,BIRC3,FAM46C,HMHA1,HLA-DOB,SIRPG,RAC2,PARP15,NLRC5,FLJ32255,IPCEF1,CD1C,PSTPIP1,MFNG,PPP2R2B,AMICA1,ANKRD44,AIM2,PTPN7,TNFSF8,TNFRSF17,ADAM28,HCP5,MBNL1,ST8SIA4,GPR114,ABCB1,HOPX,ZAP70,IGKC,CYFIP2,TNFRSF1B,CXorf65,OSBPL3,CCR6,IL21R,KLRC3,IL12RB1,ARHGAP30,ITPR1,FGD3,UBASH3A,SH2D2A,MAL,FCRL5,ATP8A1,LCP1,PBX4,PREX1,KBTBD8,SERPINB9,TRG-AS1,CLIC5,IGLL3P,GVINP1,LPXN,PCED1B,CAMK4,SLA2,BANK1,ITGAL,RCSD1,IGHD,KLRF1,CCR5,ICAM3,SH2D1B,APOBEC3G,PTPRCAP,LAMP3,AKNA,SLA,CD53,THEMIS,TOX2,LINC01215,EVI2B,CD3G,KLHL6,P2RX5,LRMP,CCL5,MAP4K1,PAX5,JAK3,TBC1D10C,BTLA,CTSW,TRDV3,TOX,PYHIN1,CD52,NAPSB,IL18RAP,CST7,GIMAP7,TMEM71,CRTAM,CCR2,MYBL1,TIGIT,SASH3,P2RY10,BCL11B,LCK,CCR7,IGHM,ZBED2,IL2RG,RUNX3,CD28,CTA-250D10.23,STAT4,ARHGAP9,PLCXD2,CD3D,SAMD3,EOMES,LTB,CD247,PPP1R16B,CD27,RHOH,SELL,ZC3H12D,IL7R,CD96,TRAT1,FCRL3,IGLC1,C16orf54,PVRIG,P2RY8,GZMH,CORO1A,KLRB1,NLRC3,CYTIP,IL2RB,CD8A,ICOS,GPR171,RASGRP1,NKG7,GPR18,ITK,MS4A1,GZMK,GNLY,IFNG,PLAC8,CD2,GZMB,TRBC1,GZMA

Supplementary Table 5. Correlation analysis of the expression of NR2F1-AS1, NR2F1, ΔNp63 and MIR205HG in TCGA pan-cancer cohort (*n* = 9186 patients)

Pearson' r	*NR2F1-AS1*	*NR2F1*	*ΔNp63*	*MIR205HG*
NR2F1-AS1	1	0.8067	-0.0255	-0.0799
NR2F1		1	-0.1681	-0.2375
ΔNp63			1	0.7769
miR205HG				1
P-value	*NR2F1-AS1*	*NR2F1*	*ΔNp63*	*MIR205HG*
NR2F1-AS1	NA	< 2.2e-16	0.0144	1.639E-14
NR2F1		NA	< 2.2e-16	< 2.2e-16
ΔNp63			NA	< 2.2e-16
miR205HG				NA

**Supplementary Table 6. Oligonucleotides used in the study**		
Usage	Name	Sequence (5'-3')
For knockdown	shNR2F1-AS1 #1-S	gatctccGCAACAATTAAACTCAACTttcaagagaAGTTGAGTTTAATTGTTGCtttttggaaa
	shNR2F1-AS1 #1-AS	agcttttccaaaaaGCAACAATTAAACTCAACTtctcttgaaAGTTGAGTTTAATTGTTGCgga
	shNR2F1-AS1 #2-S	gatctccGTCTATGTGCCACCTTACAttcaagagaTGTAAGGTGGCACATAGACtttttggaaa
	shNR2F1-AS1 #2-AS	agcttttccaaaaaGTCTATGTGCCACCTTACAtctcttgaaTGTAAGGTGGCACATAGACgga
	shPTBP1 #1-S	ccggTGTCACTAACGGACCGTTTATctcgagATAAACGGTCCGTTAGTGACAtttttg
	shPTBP1 #1-AS	aattcaaaaaTGTCACTAACGGACCGTTTATctcgagATAAACGGTCCGTTAGTGACA
	shPTBP1 #2-S	ccggGCGTGAAGATCCTGTTCAATActcgagTATTGAACAGGATCTTCACGCtttttg
	shPTBP1 #2-AS	aattcaaaaaGCGTGAAGATCCTGTTCAATActcgagTATTGAACAGGATCTTCACGC
	siPTBP1 #1	UAUUGAACAGGAUCUUCACGC
	siPTBP1 #2	UUUAAUCGGUUCUCUCCGCGU
qPCR primers	ΔNp63-promoter-F1	TACTTGGGACCCTGAGCCTTA
	ΔNp63-promoter-R1	GGTTCTGGATGAGTCACGCT
	ΔNp63-promoter-F2	GTTAACGGGACCGGTGGTT
	ΔNp63-promoter-R2	TTTCCCCAGAGAAATGCCAGT
	ΔNp63-promoter-F3	GGAGAGGCCTCACTCCATTG
	ΔNp63-promoter-R3	CTATAGGCATGAGGAGGCGG
	NR2F1-promoter-F1	TTTGCGCGTTTATTTGGGCA
	NR2F1-promoter-R1	CATTGTACGCAGCTGATGGC
	NR2F1-promoter-F2	ATGGTGGGGGTTTCATTCCC
	NR2F1-promoter-R2	TTATGAGCGTGAAGAGAGTGT
	NR2F1-promoter-F3	TCTTGGCTCCTTGACACGAG
	NR2F1-promoter-R3	TTAAACGGGAGGACTCGCAG
	Human NR2F1-AS1-F1	TTGCCAACAGGCAAATGTCC
	Human NR2F1-AS1-R1	TGGGGAACTGTCATGCCAAA
	Human NR2F1-AS1-F2	CGCGAGGGCGTAAAAGTTTG（For cytoplasm/nucleus distribution detection）
	Human NR2F1-AS1-R2	CACCGCCATTCATCCTGGTT（For cytoplasm/nucleus distribution detection）
	Human GAPDH-F1	GAAGGTGAAGGTCGGAGTC
	Human GAPDH-R1	GAAGATGGTGATGGGATTTC
	Human GAPDH-R2	TTTTGGAGGGATCTCGCTCC（F1 and R2 were used for cytoplasm/nucleus distribution detection）
	Human U6-F	CTCGCTTCGGCAGCACA
	Human U6-R	AACGCTTCACGAATTTGCGT
	Human NR2F1-F	GCCTCAAAGCCATCGTGCTG
	Human NR2F1-R	CCTCACGTACTCCTCCAGTG
	Human ΔNp63-F	GGAAAACAATGCCCAGACTC
	Human ΔNp63-R	GTGGAATACGTCCAGGTGGC
	hsa-miR205-5P-F	CCTTCATTCCACCGGAGT
	hsa-miR205-5P-R	GTCCAGTTTTTTTTTTTTTTTCAGACT

	hsa-miR205-3P-F	CAGGATTTCAGTGGAGTGAAG
	hsa-miR205-3P-R	GGTCCAGTTTTTTTTTTTTTTTGAAC
	Human CDH1-F	GTCACTGACACCAACGATAATCC
	Human CDH1-R	TTTCAGTGTGGTGATTACGACGTTA
	Human CDH2-F	CCATCAAGCCTGTGGGAATC
	Human CDH2-R	GCAGATCGGACCGGATACTG
	Human VIM-F	CCGAAAACACCCTGCAATCTTTC
	Human VIM-R	CACATCGATTTGGACATGCTGT
	Human FN1-F	CACCACAGAAGGGCGACAG
	Human FN1-R	TGCCCTCAGAAGTGCAATCA
	Human ZEB1-F	GGCATACACCTACTCAACTACGG
	Human ZEB1-R	TGGGCGGTGTAGAATCAGAGTC
	Human ZEB2-F	AATGCACAGAGTGTGGCAAGGC
	Human ZEB2-R	CTGCTGATGTGCGAACTGTAGG
	Human SNAI1-F	TGCCCTCAAGATGCACATCCGA
	Human SNAI1-R	GGGACAGGAGAAGGGCTTCTC
	Human SNAI2-F	ATCTGCGGCAAGGCGTTTTCCA
	Human SNAI2-R	GAGCCCTCAGATTTGACCTGTC
	Human TWIST1-F	GCCAGGTACATCGACTTCCTCT
	Human TWIST1-R	TCCATCCTCCAGACCGAGAAGG
For reverse transcription of miRNA	RT-primer	CAGGTCCAGTTTTTTTTTTTTTTTTTVN; V, A/C/G; N, A/G/C/T.
For cloning the template of *in vitro* transcription	probe-F	GGTTCCAATATGGCAAAGTAC
	probe-R	ACGGCAGGTTTCATTCTAGG
	T7-F	CTCCGCCCCATTGACGCAAAT
For activating *NAS1* expression by CRISPR/Cas9 synergistic activation mediator	*NAS1*-sgRNA1-S	CACCGGGGGGTGCGAGTATGCGAT
	NAS1-sgRNA1-AS	AAACATCGCATACTCGCACCCCCC
	NAS1-sgRNA2-S	CACCGAGCGATCTCAAAACGAGGG
	NAS1-sgRNA2-AS	AAACCCCTCGTTTTGAGATCGCTC
	NAS1-sgRNA3-S	CACCGAGCGGCTCCCGTCCGCTAGG
	NAS1-sgRNA3-AS	AAACCCTAGCGGACGGGAGCCGCTC
	NAS1-sgRNA4-S	CACCGTCCTGAAGGTGATTGGTCGC
	NAS1-sgRNA4-AS	AAACGCGACCAATCACCTTCAGGAC

References

1. Jiang, Y.Z., et al. Genomic and Transcriptomic Landscape of Triple-Negative Breast Cancers: Subtypes and Treatment Strategies. Cancer cell 35, 428-440 e425 (2019).

2. Koslicki, D. & Thompson, D.J. Coding sequence density estimation via topological pressure. J Math Biol 70, 45-69 (2015).

3. Kong, L., et al. CPC: assess the protein-coding potential of transcripts using sequence features and support vector machine. Nucleic acids research 35, W345-349 (2007).

4. Lanczky, A., et al. miRpower: a web-tool to validate survival-associated miRNAs utilizing expression data from 2178 breast cancer patients. Breast cancer research and treatment 160, 439-446 (2016).
