# Supplementary material for: Long non-coding RNA NR2F1-AS1 induces breast cancer lung metastatic dormancy by regulating NR2F1 and ΔNp63
Source: Nat Commun. 2021 Sep 2;12:5232. doi: 10.1038/s41467-021-25552-0 (PMC8413371; doi:10.1038/s41467-021-25552-0)
Supplement: Supplementary file 3 — Reporting Summary [file 41467_2021_25552_MOESM3_ESM.pdf]

nature research

Corresponding author(s): Guohong Hu

Last updated by author(s): 2021/07/05

Reporting Summary

Nature Research wishes to improve the reproducibility of the work that we publish. This form provides structure for consistency and transparency in reporting. For further information on Nature Research policies, see our Editorial Policies and the Editorial Policy Checklist.

Statistics

For all statistical analyses, confirm that the following items are present in the figure legend, table legend, main text, or Methods section.

n/a	Confirmed	
☐	☒	The exact sample size (*n*) for each experimental group/condition, given as a discrete number and unit of measurement
☐	☒	A statement on whether measurements were taken from distinct samples or whether the same sample was measured repeatedly
☐	☒	The statistical test(s) used AND whether they are one- or two-sided<br>*Only common tests should be described solely by name; describe more complex techniques in the Methods section.*
☒	☐	A description of all covariates tested
☐	☒	A description of any assumptions or corrections, such as tests of normality and adjustment for multiple comparisons
☐	☒	A full description of the statistical parameters including central tendency (e.g. means) or other basic estimates (e.g. regression coefficient) AND variation (e.g. standard deviation) or associated estimates of uncertainty (e.g. confidence intervals)
☐	☒	For null hypothesis testing, the test statistic (e.g. *F*, *t*, *r*) with confidence intervals, effect sizes, degrees of freedom and *P* value noted<br>*Give P values as exact values whenever suitable.*
☒	☐	For Bayesian analysis, information on the choice of priors and Markov chain Monte Carlo settings
☒	☐	For hierarchical and complex designs, identification of the appropriate level for tests and full reporting of outcomes
☐	☒	Estimates of effect sizes (e.g. Cohen's *d*, Pearson's *r*), indicating how they were calculated

Our web collection on statistics for biologists contains articles on many of the points above.

Software and code

Policy information about availability of computer code

Data collection	Bioluminescent imaging data were collected by IndiGo version 2.0.5.0 software. Cells were sorted through summit 6.3.1 or analyzed with Gallios software 1.2.
Data analysis	GraphPad Prism 8 (GraphPad Software, USA) was used to draw graphs and analyze statistical data. FlowJo v10 (Tree Star, USA) and ModFit LT 5.0 (Verity Software House, USA) were used to analyze flow cytometry data. Immunofluorescent staining data were analyzed by ZEN software v2.3 (Zeiss, Germany). Gene set enrichment analysis (GSEA) were analyzed by gsea2-2.2.4.jar. catRAPID v1.0 and IntaRNA 2.0 were used to analyze the interaction between proteins and RNAs. ShRNAs were designed with Sfold 2.2.

For manuscripts utilizing custom algorithms or software that are central to the research but not yet described in published literature, software must be made available to editors and reviewers. We strongly encourage code deposition in a community repository (e.g. GitHub). See the Nature Research guidelines for submitting code & software for further information.

Data

Policy information about availability of data

All manuscripts must include a data availability statement. This statement should provide the following information, where applicable:

- Accession codes, unique identifiers, or web links for publicly available datasets
- A list of figures that have associated raw data
- A description of any restrictions on data availability

The RNA sequencing data of MCF10 cell lines and cancer cells with NAS1 knockdown or NR2F1 overexpression, related to Fig 2A, 6A, 6G, 7F, 7G, S1G, S9H, have been deposited in the National Omics Data Encyclopedia (https://www.biosino.org/node, ID: OEP000853, https://www.biosino.org/node/project/detail/OEP000853). It is freely accessible to all.

Field-specific reporting

Please select the one below that is the best fit for your research. If you are not sure, read the appropriate sections before making your selection.

- [x] Life sciences
- [ ] Behavioural & social sciences
- [ ] Ecological, evolutionary & environmental sciences

For a reference copy of the document with all sections, see nature.com/documents/nr-reporting-summary-flat.pdf

Life sciences study design

All studies must disclose on these points even when the disclosure is negative.

Sample size	Sample sizes for each experiment are stated in figure legends. Sample sizes are determined empirically, and similar in size to most existing studies in the same field. For in vitro assays, n =3-4. For in vivo mouse experiments, usually n = 5-9 mice were used for long-term metastasis analyses and tumor-initiating capacity studies, and n = 30 random microscopic fields from 3 mice (ref. 13 & 20) each group were used for analyzing the early stage of colonization. No statistical method was used to predetermine sample size.
Data exclusions	Usually no data were excluded, except in the in vivo mouse experiments when 1-2 mice were dropped out for further analyses due to accidental deaths of the animals.
Replication	Experiments were repeated with same conditions and obtained similar results. All attempts to reproduce data were successful. The number of repeats were indicated in figure legends.
Randomization	Mice were randomly allocated among groups. For in vitro studies, within an independent experiment, the experiment was carried out strictly in accordance with the single variable principle. For biological repeats, experimental materials (cells, etc.) are reprepared and experiments were performed on separately. Treatment groups were divided randomly and equally. Sample collection was done in a random order.
Blinding	For animal experiments, investigators were not blinded during mouse grouping since the animal experiments were performed by the same researchers for data consistency, grouping blinding was impossible. Since the mice were randomly allocated among groups, blinding should not be relevant in these analyses. Due to obvious differences between groups, many in vitro assays were not blinded. The same results have been repeated by multiple members of the research team. For experiments in which data are analyzed and collected by machines such as flow cytometry, qPCR and so on, researchers were not blinded. RNA-seq and LC/MS data collection and analysis were performed by the core facility members and the analysis was performed following the standard procedure by two research members.

Reporting for specific materials, systems and methods

We require information from authors about some types of materials, experimental systems and methods used in many studies. Here, indicate whether each material, system or method listed is relevant to your study. If you are not sure if a list item applies to your research, read the appropriate section before selecting a response.

Materials & experimental systems

n/a	Involved in the study	
[ ]	[x]	Antibodies
[ ]	[x]	Eukaryotic cell lines
[x]	[ ]	Palaeontology and archaeology
[ ]	[x]	Animals and other organisms
[ ]	[x]	Human research participants
[x]	[ ]	Clinical data
[x]	[ ]	Dual use research of concern

Methods

n/a	Involved in the study	
[x]	[ ]	ChIP-seq
[ ]	[x]	Flow cytometry
[x]	[ ]	MRI-based neuroimaging

Antibodies

Antibodies used	APC mouse anti-human CD24 (Biolegend, 311118),<br>FITC mouse anti-human CD44 (BD Pharmingen, 555478),<br>PE mouse anti-human CD44 (BD Pharmingen, 555479),<br>chicken polyclonal GFP antibody (Abcam, ab13970),<br>Anti-GFP antibody (Abcam, ab290),<br>donkey anti-Chicken IgY (FITC) secondary antibody (Invitrogen, SA172000),<br>E-Cadherin (24E10) rabbit mAb (Cell Signaling technology, 3195S),<br>N-Cadherin mouse antibody (BD Pharmingen, 610920),<br>Fibronectin mouse antibody (Santa Cruz, sc-59826),<br>Vimentin (D21H3) XP® rabbit mAb (Cell Signaling technology, 5741S),<br>ZEB1 rabbit polyclonal antibody (proteintech, 21544-1-AP),<br>mouse anti-Twist antibody (Abcam, ab50887),

Alexa Fluor 488 donkey anti-rabbit IgG (Invitrogen, A-21206),
Alexa Fluor 555 donkey anti-mouse IgG (Invitrogen, A31570),
rabbit anti-human GAPDH (Merck/Millipore, SAB2103104),
rabbit anti-COUP TF1 antibody (Abcam, ab181137),
p63-α (D2K8X) XP® rabbit mAb (Cell Signaling technology, 13109S),
PTBP1 (E4I3Q) rabbit mAb (Cell Signaling technology, 57246),
Lamin A/C rabbit polyclonal antibody (proteintech, cat#10298-1-AP),
rabbit anti-HA (Cell Signaling technology, 3724S),
HRP-conjugated Goat Anti-mouse IgG (Merck/Millipore, 401215),
HRP-conjugated Goat Anti-rabbit IgG (Merck /Millipore, 401315),
normal rabbit IgG (Cell Signaling technology, 2729S).

Validation

All antibodies were validated according to respective manufacturer's information. Anti-GFP antibody (Abcam, ab290) for RIP was validated with IgG control (fig. 5e) and GFP-MS2-free negative control (not show). rabbit anti-HA (Cell Signaling technology, 3724S) for RIP was validated with IgG control (fig. 5b&d) and HA-tag-free negative control (not show). ΔNp63 detected with p63-α (D2K8X) XP® rabbit mAb (Cell Signaling technology, 13109S) was validated with ΔNp63-knockdown assay (fig. 6e), and manufacturer also stated that this antibody could detect ΔNp63.
APC mouse anti-human CD24 (Biolegend, 311118), Human, FC, https://www.biolegend.com/en-us/products/apc-anti-human-cd24-antibody-6125?GroupID=BLG5916
FITC mouse anti-human CD44 (BD Pharmingen, 555478), Human, FC, https://www.bdbiosciences.com/en-us/products/reagents/flow-cytometry-reagents/research-reagents/single-color-antibodies-ruo/fitc-mouse-anti-human-cd44.555478
PE mouse anti-human CD44 (BD Pharmingen, 555479), Human, FC, https://www.bdbiosciences.com/en-us/products/reagents/flow-cytometry-reagents/research-reagents/single-color-antibodies-ruo/pe-mouse-anti-human-cd44.555479
chicken polyclonal GFP antibody (Abcam, ab13970), ,WB ICC/IF, https://www.abcam.com/GFP-antibody-ab13970.html
Anti-GFP antibody (Abcam, ab290),, ELISA IHC-FrFl Electron Microscopy IHC-FoFr ICC IHC-P IHC-Fr IP WB, https://www.abcam.com/GFP-antibody-ab290.html
donkey anti-Chicken IgY (FITC) secondary antibody (Invitrogen, SA172000), Chicken, IHC ICC/IF, https://www.thermofisher.cn/cn/zh/antibody/product/Donkey-anti-Chicken-IgY-H-L-Secondary-Antibody-Polyclonal/SA1-72000?adobe_mc=MCMID%7C89186774675130844752397467780430890508%7CMCAID%3D3067FB3C5E0F5586-600000CC30478EB7%7CMCORGID%3D5B135A0C5370E6B40A490D44%40AdobeOrg%7CTS=1614293705
E-Cadherin (24E10) rabbit mAb (Cell Signaling technology, 3195S), Human Mouse, WB IHC IF FC,https://www.cellsignal.com/products/primary-antibodies/e-cadherin-24e10-rabbit-mab/3195
N-Cadherin mouse antibody (BD Pharmingen, 610920), Human Mouse Rat Chicken,WB IF IP, https://www.bdbiosciences.com/en-us/products/reagents/microscopy-imaging-reagents/immunofluorescence-reagents/purified-mouse-anti-n-cadherin.610921
Fibronectin mouse antibody (Santa Cruz, sc-59826),
Vimentin (D21H3) XP® rabbit mAb (Cell Signaling technology, 5741S), Human Mouse Rat Monkey, WB IHC IF FC, https://www.cellsignal.com/products/primary-antibodies/vimentin-d21h3-xp-rabbit-mab/5741
ZEB1 rabbit polyclonal antibody (proteintech, 21544-1-AP), Human Mouse Rat, WB IP IHC IF CoIP ChIP ELISA, https://www.ptglab.com/Products/ZEB1-Antibody-21544-1-AP.htm
mouse anti-Twist antibody (Abcam, ab50887),Human Mouse Rat,WB ICC, https://www.abcam.com/Twist-antibody-Twist2C1a-ab50887.html
Alexa Fluor 488 donkey anti-rabbit IgG (Invitrogen, A-21206), Rabbit, IHC ICC/IF FC, https://www.thermofisher.cn/cn/zh/antibody/product/Donkey-anti-Rabbit-IgG-H-L-Highly-Cross-Adsorbed-Secondary-Antibody-Polyclonal/A-21206?adobe_mc=MCMID%7C89186774675130844752397467780430890508%7CMCAID%3D3067FB3C5E0F5586-600000CC30478EB7%7CMCORGID%3D5B135A0C5370E6B40A490D44@AdobeOrg%7CTS%3D1614293705
Alexa Fluor 555 donkey anti-mouse IgG (Invitrogen, A31570), Mouse, IHC ICC/IF, https://www.thermofisher.cn/cn/zh/antibody/product/Donkey-anti-Mouse-IgG-H-L-Highly-Cross-Adsorbed-Secondary-Antibody-Polyclonal/A-31570?adobe_mc=MCMID%7C89186774675130844752397467780430890508%7CMCAID%3D3067FB3C5E0F5586-600000CC30478EB7%7CMCORGID%3D5B135A0C5370E6B40A490D44@AdobeOrg%7CTS%3D1614293705
rabbit anti-human GAPDH (Merck/Millipore, SAB2103104), Human Horse Pig Mouse Rabbit Rat, WB, https://www.sigmaaldrich.cn/CN/zh/product/sigma/sab2103104?context=product
rabbit anti-COUP TF1 antibody (Abcam, ab181137), Mouse Rat Human, WB ICC/IF FC, https://www.abcam.com/coup-tf1-antibody-epr10841-ab181137.html
p63-α (D2K8X) XP® rabbit mAb (Cell Signaling technology, 13109S), Human, WB IP IF FC ChIP, https://www.cellsignal.com/products/primary-antibodies/p63-a-d2k8x-xp-rabbit-mab/13109
PTBP1 (E4I3Q) rabbit mAb (Cell Signaling technology, 57246), Human Mouse Rat Monkey, https://www.cellsignal.com/products/primary-antibodies/ptbp1-e4i3q-rabbit-mab/57246
Lamin A/C rabbit polyclonal antibody (proteintech, cat#10298-1-AP), Human Mouse Rat, WB IP IF FC ELISA, https://www.ptglab.com/Products/lamin-A-Antibody-10298-1-AP.htm
rabbit anti-HA (Cell Signaling technology, 3724S), , WB IP IHC IF FC ChIP, https://www.cellsignal.com/products/primary-antibodies/ha-tag-c29f4-rabbit-mab/3724
HRP-conjugated Goat Anti-mouse IgG (Merck/Millipore, 401215), ELISA Immunoelectrophoresis, https://www.merckmillipore.com/CN/zh/product/Goat-Anti-Mouse-IgG-HL-Chain-Specific-Peroxidase-Conjugate,EMD_BIO-401215
HRP-conjugated Goat Anti-rabbit IgG (Merck /Millipore, 401315), ELISA Immunoelectrophoresis, https://www.merckmillipore.com/CN/zh/product/Goat-Anti-Rabbit-IgG-H-L-Chain-Specific-Peroxidase-Conjugate,EMD_BIO-401315?ReferrerURL=https%3A%2F%2Fcn.bing.com%2F
normal rabbit IgG (Cell Signaling technology, 2729S), none, IP ChIP, https://www.cellsignal.com/products/primary-antibodies/normal-rabbit-igg/2729

Eukaryotic cell lines

Policy information about cell lines

Cell line source(s)	HeLa and MCF7 were purchased from from the Cell Bank, Chinese Academy of Sciences. MDA-MB-231 and its derivatives were obtained from Dr. Massague (MSKCC), who established the derivatives from the parental cell line MDA-MB-231 obtained from ATCC. MCF10AT, MCF10CA1h and MCF10CA1a cell lines were obtained from Dr. Miller (Wayne State University) who established them (American Journal of Pathology 1996;148(1):313-319; Cancer Res 1992; 52:1399-1405).
Authentication	Cell lines were authenticated via STR genotyping.
Mycoplasma contamination	All cell lines were tested negative for mycoplasma contamination.
Commonly misidentified lines (See ICLAC register)	No cell lines adopted in this study is listed in the database of commonly misidentified cell lines maintained by ICLAC.

Animals and other organisms

Policy information about studies involving animals; ARRIVE guidelines recommended for reporting animal research

Laboratory animals	Female NOD/SCID mice and BALB/c nude mice aged 6-8 weeks were used in all animal studies. Keep in the standard SPF grade animal house. Mice were housed in a 12h light/dark cycle (6:00-18:00 light and 18:00-6:00 dark), with controlled room temperature at 24±2 °C and relative humidity at 40-50%.
Wild animals	The study did not involve wild animals.
Field-collected samples	The study did not involve filed-collected samples.
Ethics oversight	All animal studies were conducted according to the guidelines for the care and use of laboratory animals and were approved by Institutional Biomedical Research Ethics Committee of Shanghai Institute of Nutrition and Health.

Note that full information on the approval of the study protocol must also be provided in the manuscript.

Human research participants

Policy information about studies involving human research participants

Population characteristics	Breast cancer tissues (female patients, age at 36-73 years) for the correlation analysis of NR2F1-AS1 and NR2F1 were obtained from the clinical sample database of the Shanghai Institute of Nutrition and Health, Chinese Academy of Sciences. Tumors tissue samples and prognostic information from female patients (age at 29-74 years) diagnosed with breast cancer and treated in Qilu Hospital of Shandong University were used in the metastasis-free and relapse-free survival analyses and tissue microarray.
Recruitment	Samples and prognostic information were obtained with informed patient consent. Samples were randomly selected, there is no potential self-selection bias or other biases. Patients treated in the year of 2006-2008 in Qilu Hospital of Shandong University with available freshly frozen or paraffin-embedded samples and sufficient prognostic information were included.
Ethics oversight	Samples and prognostic information were obtained with informed patient consent and the approval from Research Review Boards of Insititute of Nutrition and Health and Qilu hospital of Shandong University.

Note that full information on the approval of the study protocol must also be provided in the manuscript.

Flow Cytometry

Plots

Confirm that:

- [x] The axis labels state the marker and fluorochrome used (e.g. CD4-FITC).
- [x] The axis scales are clearly visible. Include numbers along axes only for bottom left plot of group (a 'group' is an analysis of identical markers).
- [x] All plots are contour plots with outliers or pseudocolor plots.
- [x] A numerical value for number of cells or percentage (with statistics) is provided.

Methodology

Sample preparation	Cancer cells harvested from culture during the logarithmic growth period were resuspended in PBS.
Instrument	MoFlo Astrios EQ Flow Cytometer (Beckman) for sorting and Gallios Analyzer (Beckman) for analysis.

Software	Collection: Summit6.3.1 for sorting and Gallios Software for analysis.<br>Data processing: FlowJo v10 (Tree Star, USA) and ModFit LT (Verity Software House, USA).
Cell population abundance	Purity of post-sort fractions are measured by flow for CD44highCD24- or CD44medCD24-, and purity is >98%,respectively.
Gating strategy	Starting cells were gated on a linear FSC/SSC plot, and further analyses were shown in all flow figures. Positive/negative populations were determined by fluorescence minus one (FMO) controls.

☒ Tick this box to confirm that a figure exemplifying the gating strategy is provided in the Supplementary Information.
